# XBP1s Mediates Cross‐resistance to Combination Treatment of CDK4/6 Inhibitors plus Endocrine Therapy in Breast Cancer

**DOI:** 10.1002/advs.202409588

**Published:** 2025-09-12

**Authors:** Yuting Sang, Shiyang Liu, Xujie Zhou, Weiru Chi, Min Xiong, Ming Chen, Hengyu Ren, Douwaner Liu, Liyi Zhang, Jingyan Xue, Yayun Chi, Jiong Wu

**Affiliations:** ^1^ Department of Breast Surgery Key Laboratory of Breast Cancer in Shanghai Fudan University Shanghai Cancer Center Shanghai 200030 China; ^2^ Department of Oncology Shanghai Medical College Fudan University Shanghai 200030 China

**Keywords:** breast cancer, CDK4/6 inhibitors, drug resistance, endocrine therapy, XBP1s

## Abstract

CDK4/6 inhibitors combined with endocrine therapy is the standard treatment for patients with hormone receptor‐positive (HR+)/human epidermal growth factor receptor 2‐negative (HER2−) metastatic breast cancer (MBC). However, the inevitable development of treatment resistance and lack of approved biomarkers for predicting therapeutic efficacy remain urgent concerns. This study indicates that XBP1 is prominently and specifically expressed in HR+/HER2− breast cancer, and correlated with unfavorable response and poor progression‐free survival in patients with MBC receiving the combined therapy. XBP1s, a transcriptionally active spliced form of XBP1, accelerates tumor progression by facilitating cell proliferation and G1/S transition, and attenuates the efficacy of palbociclib and fulvestrant. Conversely, it can be reversed through epigenetic and pharmacological inhibition of XBP1s expression. Mechanistically, XBP1s activates the E2F1 pathway and upregulates downstream targets by transcriptionally activating SND1. Using patient‐derived organoids, it is confirmed that XBP1s plays a pro‐survival role and counteracts E2F1 pathway inhibition caused by the combined therapy, whereas 4µ8C sensitizes cells and exerts a synergistic effect with both fulvestrant and palbociclib. In conclusion, the findings indicate that XBP1s may serve as a potential marker for identifying patients who may not benefit from the medication, and offer a novel therapeutic strategy for patients with HR+/HER2− MBC.

## Graphical abstract

Elevated spliced form of X‐box–binding protein 1 (XBP1) correlates with unfavorable responses to endocrine therapy plus CDK4/6 inhibitors in HR+/HER2− advanced breast cancer. XBP1s facilitates cell proliferation and G1/S transition by transcriptionally activating SND1, thereby activating the E2F1 pathway. By blocking XBP1 splicing, 4µ8C sensitizes cells and exhibits a synergistic effect with fulvestrant and palbociclib, providing a novel therapeutic strategy.

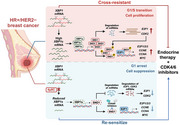

## 1 Introduction

Hormone receptor‐positive (HR+)/human epidermal growth factor receptor 2‐negative (HER2−) breast cancer is the most common subtype, comprising 60% of all metastatic breast cancer (MBC).^[^ ^1^ ^]^ Given that the development and progression of HR+/HER2− breast cancer is mainly driven by estrogen receptor (ER) signaling, endocrine therapy constitutes the cornerstone of systemic treatment of this subtype.^[^ ^2^ ^]^ Over the last decade, extensive crosstalk between ER signaling and cyclin‐dependent kinases (CDK)4/6–cyclin D axis, and the fundamental role of CDK4/6–cyclin D axis in cell proliferation and endocrine resistance were identified in HR+/HER2− breast cancer.^[^ ^3^ ^]^ During the G1/S cell‐cycle transition, CDK4/6–cyclin D complex phosphorylates retinoblastoma (Rb), causing the release of E2F and activation of E2F‐driven transcription, which leads to transcriptional activation of a series of genes required for the entry into S phase.^[^ ^4^ ^]^ Accordingly, CDK4/6 inhibitors block this pathway, restrict the G1/S transition, and disrupt the uncontrolled cell proliferation in tumor cells. Also, laboratory‐based and clinical studies have revealed that the addition of CDK4/6 inhibitors to endocrine therapy results in synergistic effects and significantly improved progression‐free survival and overall survival in patients with advanced HR+/HER2− breast cancer.^[^ ^5^ ^]^ Thus, CDK4/6 inhibitors in combination with endocrine therapy has become the standard therapeutic approach for patients with HR+/HER2− MBC.^[^ ^6^ ^]^ Nevertheless, resistance is inevitable in the advanced setting. Some patients do not respond to the combined CDK4/6 inhibitors and endocrine therapy, while others experience disease progression during the treatment, which remains a major obstacle.^[^ ^7^ ^]^ Therefore, there is an urgent clinical need to identify predictive biomarkers and resistance mechanisms to the combined treatment of CDK4/6 inhibitors and endocrine therapy.

Activation of the unfolded protein response (UPR) is a hallmark event in tumor cells, which serves as a pivotal mechanism for cell survival under the high‐pressure microenvironments caused by rapid proliferation, such as nutrient deficiency, hypoxia, and acid‐base disorders.^[^ ^8^ ^]^ The inositol‐requiring enzyme 1α (IRE1α)–X‐box–binding protein 1 (XBP1) pathway is the most conserved UPR signaling. Following UPR induction, IRE1α phosphorylates and activates the RNase domain, which cleaves and removes a 26‐nucleotide intron from *XBP1* mRNA, generating spliced XBP1 (XBP1s).^[^ ^9^ ^]^ As unspliced XBP1 (XBP1u) lacks functional transactivation domains, it has been generally believed that XBP1s serves as a dominant positive variant of XBP1 with transcriptional function.^[^ ^10^ ^]^ Previous studies have shown that activation of the IRE1α–XBP1 pathway mediates cell stemness, migration, angiogenesis, and treatment resistance in breast cancer.^[^ ^11^ ^]^ For example, XBP1s has been reported to regulate hypoxia‐responsive genes and contribute to tumorigenicity and progression in triple‐negative breast cancer.^[^ ^12^ ^]^ Hu et al. indicated a pro‐survival role for XBP1s through modulation of the nuclear factor‐kappa B (NF‐κB) pathway in breast cancer.^[^ ^13^ ^]^ In addition, a positive regulatory feedback loop was identified between XBP1s and ER signaling.^[^ ^14^ ^]^ Therefore, XBP1s has received extensive attention and is considered a promising therapeutic target in breast cancer.

In this study, we demonstrated significantly elevated levels of *XBP1* and *XBP1s* in HR+/HER2− breast cancer, which were associated with an unfavorable therapeutic response and poor prognosis in patients who received the combination treatment of CDK4/6 inhibitors plus endocrine therapy. Notably, we discovered that XBP1s promoted cell proliferation and G1/S transition, and impaired the efficiency of CDK4/6 inhibitors and endocrine therapy. Using patient‐derived organoid models, we further verified that 8‐formyl‐7‐hydroxy‐4‐methylcoumarin (4µ8C), a selective IRE1α RNase inhibitor, could block XBP1 splicing, sensitize cells to the treatment, and exert a synergistic effect with both fulvestrant and palbociclib. Mechanically, XBP1s bypass the CDK4/6–cyclin D–Rb pathway through transcriptional activation of staphylococcal nuclease domain‐containing protein 1 (SND1), which triggers activation of the E2F transcription factor 1 (E2F1) pathway, leading to upregulation of downstream target genes and cell cycle transit. Altogether, our results indicated that XBP1s may serve as a prospective prognostic marker for the efficiency of CDK4/6 inhibitors in combination with endocrine therapy, and a potential therapeutic target for delaying the onset of resistance in HR+/ HER2− MBC.

## 2 Results

### 2.1 XBP1s Correlated with Resistance to the Combination Treatment of Endocrine Therapy plus CDK4/6 Inhibitors in Patients with Metastatic HR+/HER2− Breast Cancer

**Figure 1:**
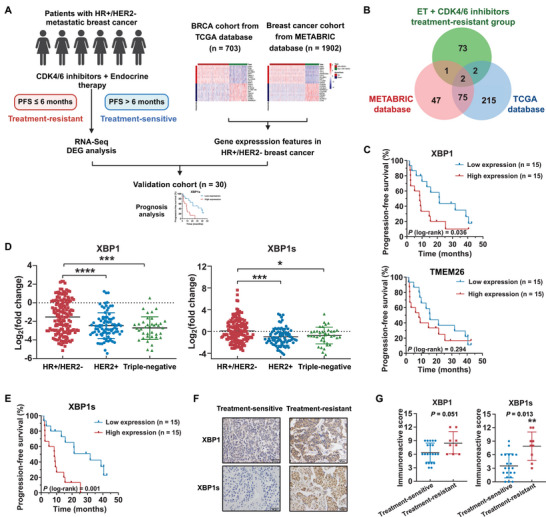
Expression levels of XBP1 and XBP1s are positively correlated with resistance to CDK4/6 inhibitors and endocrine therapy in patients with HR+/HER2− metastatic breast cancer. A) Schematic for the study design and analytical workflow. PFS, progression‐free survival; RNA‐seq, RNA sequencing; DEG, differentially expressed gene; BRCA, breast invasive carcinoma. B) Venn diagram showing the shared numbers and overlaps of upregulated genes identified in HR+/HER2− breast cancer tumors from the TCGA and METABRIC datasets, and the treatment‐resistant cohort of patients with HR+/HER2− metastatic breast cancer. ET, endocrine therapy. C) Kaplan–Meier curves of progression‐free survival (PFS) in the validation cohort of patients with high and low levels of *XBP1* and *TMEM26* expressions based on qRT‐PCR assay. *P*‐values are indicated and were calculated using the log‐rank test. D) *XBP1* and *XBP1s* expression levels in primary breast tumors of different molecular subtypes (HR+/HER2− subtype, *n* = 152; HER2+ subtype, *n* = 84; Triple‐negative subtype, *n* = 38). *p < 0.05, ***p < 0.001, ****p < 0.0001. E) Kaplan–Meier curves of PFS in the validation cohort of patients with high and low levels of *XBP1s* expression. *P*‐values are indicated and were calculated using the log‐rank test. F) Immunohistochemistry (IHC) staining for XBP1s and XBP1 in representative tumor samples from patients who were sensitive (PFS > 6 months) or resistant (PFS ≤ 6 months) to combination therapy of CDK4/6 inhibitors and endocrine therapy. Scale bar, 50 µm. G) The immunoreactive score of IHC staining for XBP1s and XBP1 in tumors from the validation cohort of patients. **p < 0.01. For statistical analysis, the log‐rank test was employed in (C) and (E); one‐way ANOVA with Tukey's post hoc test was utilized for (D); the Mann–Whitney *U* test was employed in (G). Data are represented as the mean ± standard deviation.

Despite significantly improved outcomes in patients with HR+/HER2− breast cancer after treatment with CDK4/6 inhibitors combined with endocrine therapy, drug resistance remains a major therapeutic obstacle. Notably, 10%–30% of patients with advanced HR‐positive breast cancer exhibit rapid resistance, while nearly half of the patients experienced tumor progression after 12 months of treatment.^[^ ^15^ ^]^ This high prevalence underscores the critical need to identify resistance mechanisms specific to the HR+/HER2− subtype to optimize patient stratification and develop targeted interventions. To mitigate confounding inherent to subtypes and pinpoint bona fide resistance drivers within this therapeutic context, we employed an intersectional strategy. We prioritized genes that showed increased expression in treatment‐resistant tumors and exhibited elevated expression in HR+/HER2− breast cancer compared to other molecular subtypes. First, we retrospectively collected the biopsy samples of metastatic sites from six patients with HR+/HER2− MBC treated with the combined therapy, and subjected them to RNA sequencing (RNA‐seq). Three patients experienced cancer progression within 6 months after administration, while the others were still being treated at the time of follow‐up. Accordingly, patients were divided into treatment‐sensitive and treatment‐resistant cohorts (**Table** **1**), and genes that were upregulated in the treatment‐resistant cohort were identified. Next, the genes exhibiting significantly higher expression in HR+/HER2− breast cancer relative to other molecular subtypes were identified by analyzing the Molecular Taxonomy of Breast Cancer International Consortium (METABRIC) and The Cancer Genome Atlas (TCGA) databases. Notably, XBP1 and transmembrane protein 26 (TMEM26), both upregulated in the treatment‐resistant cohort, exhibited high expression levels in HR+/HER2− breast tumors compared with other subtypes (**Figure** **1**A,B), suggesting their potential association with resistance to endocrine therapy plus CDK4/6 inhibitors in this subtype. In addition, gene set enrichment analysis (GSEA) showed that the E2F targets, IL6‐JAK‐STAT signaling, KRAS signaling, MYC targets, and PI3K‐AKT‐MTOR signaling were significantly enriched in treatment‐resistant tumors (Figure S1A, Supporting Information). To verify the clinical relevance of the candidate genes, quantitative real‐time PCR (qRT‐PCR) was used to detect the expression levels of *XBP1* and *TMEM26* in a validation cohort of 30 patients with HR+/HER2− MBC who were treated with the combined therapy. Kaplan–Meier analysis showed that patients with higher expression levels of *XBP1* had worse progression‐free survival (PFS), while no significant association was observed between *TMEM26* expression and PFS (Figure 1C). Upon analyzing RNA‐seq data from the TCGA database using the GEPIA, we observed that *XBP1* expression was predominantly restricted to breast tissue and significantly upregulated in tumor samples (Figure S1B, Supporting Information). In contrast, *TMEM26* was highly expressed in normal breast tissue (Figure S1C, Supporting Information). Moreover, we confirmed the elevated expression level of XBP1 by analyzing and intersecting RNA‐seq data from endocrine‐resistant or CDK4/6 inhibitor‐resistant cohorts (GSE224435, GSE229146, and GSE229235) with our discovery cohort (Figure S1D, Supporting Information).^[^ ^16^ ^]^ Therefore, *XBP1* was selected for further investigation.

**Table 1:** Patients information of discovery cohort.

ID	Age	Receptor status	Adjuvant therapy	Metastatic therapy	Therapeutic response (≤6 cycles)
1	55	ER+/PR+/HER2−	AC, AI	Fulvestrant+CDK4/6 inhibitor^a)^	PD
2	59	ER+/PR+/HER2−	AC‐T, AI	Fulvestrant+CDK4/6 inhibitor	PD
3	63	ER+/PR+/HER2−	AI	Fulvestrant+CDK4/6 inhibitor	PD
4	69	ER+/PR+/HER2−	AC, AI	Fulvestrant+CDK4/6 inhibitor	PR
5	65	ER+/PR+/HER2−	AI	Fulvestrant+CDK4/6 inhibitor	SD
6	69	ER+/PR+/HER2−	None (*de novo* metastatic)	Fulvestrant+CDK4/6 inhibitor	SD

^a)^ CDK4/6 inhibitors include Palbociclib and Abemaciclib.

*AC*, doxorubicin/cyclophosphamide; *AC‐T*, AC followed by docetaxel; *AI*, aromatase inhibitors; *PR*, partial response; *SD*, stable disease; *PD*, progressive disease.

Of note, XBP1 could generate the transcriptionally active isoform XBP1s through activated IRE1α, which initiates a translational frame‐shift process following removal of a 26‐base intron from *XBP1* mRNA. Although XBP1 and XBP1s share extensive sequence homology, XBP1s acquires unique C‐terminal transcriptional domains, which enable it to function as a potent transcription factor and a critical effector of the IRE1α–XBP1 pathway.^[^ ^9^ ^]^ Thus, we designed junction‐targeting primers for XBP1s to quantify via qRT‐PCR and assessed the expression levels of XBP1 and XBP1s in early breast cancer tissues. The results showed that both *XBP1* and *XBP1s* exhibited high expression levels in HR+/HER2− breast tumors (Figure 1D). Western blot assay also indicated a slightly elevated trend of XBP1 and XBP1s expression in HR+/HER2− breast cancer cell lines (Figure S1E, Supporting Information). However, Kaplan–Meier analysis showed no statistically significant correlation between XBP1 expression levels and overall survival (OS) in HR+/HER2− breast cancer from the TCGA and METABRIC datasets, as well as our cohort of patients with HR+/HER2− early breast cancer (Figure S1F–H, Supporting Information). Whereas the high expression levels of XBP1s exhibited a significant positive correlation with worse OS in HR+/HER2− early breast cancer (Figure S1H, Supporting Information). Notably, elevated *XBP1s* level was significantly associated with unfavorable PFS outcomes in patients with HR+/HER2− MBC who received the combination therapy (Figure 1E). In addition, we quantified the XBP1 and XBP1s levels in tumors from the validation cohort using immunohistochemistry (IHC) scores (Figure 1F; Figure S1I, Supporting Information). The tumors from patients in the treatment‐resistant group had significantly higher IHC scores of XBP1s than those from treatment‐sensitive patients. However, although XBP1 immunoreactivity scores showed a strong trend toward elevation in treatment‐resistant specimens, statistical significance was not reached (Figure 1G). Collectively, these data indicated that *XBP1s* is highly expressed in HR+/HER2− breast tumors and exhibits a significant correlation with poor prognosis and resistance to the combination of CDK4/6 inhibitors and endocrine therapy in these patients.

**Figure 2:**
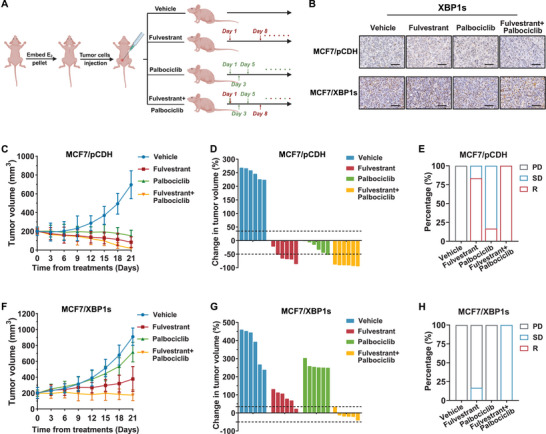
XBP1s attenuated the therapeutic efficacy of CDK4/6 inhibitors and endocrine therapy in vivo. A) Schematic diagram illustrating the workflow of the in vivo study. B–H) Mice with cell‐derived xenografts of MCF7/pCDH or MCF7/XBP1s were randomly divided into four treatment groups: vehicle, palbociclib, fulvestrant, or their combination. B) Representative images of IHC staining for XBP1s in MCF7/pCDH and MCF7/XBP1s cell‐derived xenografts from different treatment groups. Scale bar, 50 µm. C) Tumor growth curve plots of MCF7/pCDH tumor in each treatment group after treatment administration. *n* = 6 per treatment group. Each data point represents the mean tumor volume ± standard deviation. D) Waterfall plot showing the change in tumor volume of individual MCF7/pCDH cell‐derived xenografts at the end of the experiment compared with the baseline tumor volume on day 0 of the treatment. *n* = 6 per treatment group. E) Percentage of tumors exhibiting different drug responses (PD, SD, R) in MCF7/pCDH cell‐derived xenografts. PD, progression disease; SD, stable disease; R, regression. F) Tumor growth curve plots of MCF7/XBP1s tumor in each treatment group after treatment administration. *n* = 6 per treatment group. Each data point represents the mean tumor volume ± standard deviation. G) Waterfall plot showing the change in tumor volume of individual MCF7/ XBP1s cell‐derived xenografts at the end of the experiment compared with the baseline tumor volume on day 0 of the treatment. *n* = 6 per treatment group. H) Percentage of tumors exhibiting different drug responses (PD, SD, R) in MCF7/XBP1s cell‐derived xenografts.

To further confirm the role of XBP1s in tumor response to CDK4/6 inhibitors plus endocrine therapy, we generated cell‐derived xenograft models using *XBP1s‐overexpressing* and control MCF7 cells. Once the tumor size reached an average volume of 200 mm^3^, the mice were randomly divided into four groups and treated with the vehicle or the indicated drugs (**Figure** **2**A). None of the treatment group mice experienced weight loss exceeding 10%, indicating tolerability of palbociclib, fulvestrant, or their combination (Figure S2A,B, Supporting Information). Next, the elevated XBP1s levels in *XBP1s*‐overexpressing tumors were validated with IHC assays, and a corresponding rise in the proportion of XBP1s‐positive cells in the medicated group, suggesting that XBP1s‐positive cells exhibited resistance to palbociclib and fulvestrant treatment (Figure 2B; Figure S2C, Supporting Information). In the MCF7/pCDH xenograft model, the individual administration of palbociclib and fulvestrant caused a significant decrease in tumor development, and a more pronounced efficacy in tumor regression was observed in the combined group (Figure 2C,D; Figure S2D,E, Supporting Information). Consequently, all xenografts in the MCF7/pCDH group exhibited a positive response to palbociclib, fulvestrant, and the combined therapy, resulting in either stable disease or regression (Figure 2E). However, *XBP1s*‐overexpressing tumors did not regress when treated with palbociclib, fulvestrant, or their combined therapy (Figure 2F–H; Figure S2F,G, Supporting Information), confirming that XBP1s overexpression confers significant resistance against palbociclib, fulvestrant, and their combination in vivo, in stark contrast to the drug‐sensitive MCF7/pCDH control group.

### 2.2 XBP1s Reduced the Responsiveness of HR+/HER2− Breast Cancer to Palbociclib, Fulvestrant, and the Combined Therapy

**Figure 3:**
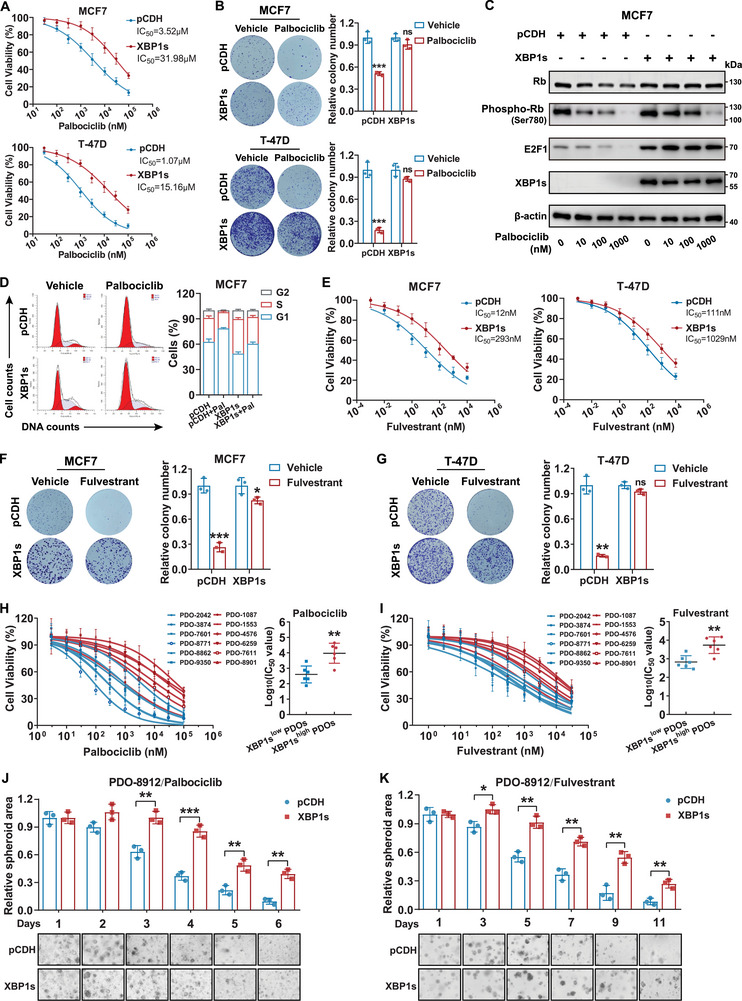
Overexpression of XBP1s confers resistance to fulvestrant and palbociclib and impairs the synergistic effect of palbociclib combined with fulvestrant in vitro. A) The dose‐response curves and half‐maximal inhibitory concentration (IC_50_) values of palbociclib in MCF7 and T‐47D cells transfected with the control or XBP1s‐overexpressing plasmid after being treated for 96 h. B) Representative images and quantification of colony formation assay in MCF7 and T‐47D cells treated with vehicle or palbociclib (3.5 × 10^−6^ m for MCF7 cells, 1 × 10^−6^ m for T‐47D cells). ns, not significant; ***p < 0.001. C) Western blotting of the indicated proteins in MCF7 cells with or without *XBP1s* overexpression after being treated with the indicated concentration of palbociclib. D) Cell cycle distribution of MCF7 cells with or without *XBP1s* overexpression after being treated with palbociclib (3.5 × 10^−6^ m). E) The dose‐response curves and IC_50_ values of fulvestrant in MCF7 and T‐47D cells transfected with the control or *XBP1s*‐overexpressing plasmid after being treated for 96 h. F) Representative images and quantification of colony formation assay after treatment with vehicle or fulvestrant in MCF7 (15 × 10^−9^ m) cells with or without *XBP1s* overexpression. *p < 0.05, ***p < 0.001. G) Representative images and quantification of colony formation assay after treatment with vehicle or fulvestrant in T‐47D cells (120 × 10^−9^ m) with or without *XBP1s* overexpression. ns, not significant; **p < 0.01. H) The dose‐response curves and IC_50_ values of palbociclib in patient‐derived organoids (PDOs) after being treated for 96 h. **p < 0.01. I) The dose–response curves and IC_50_ values of palbociclib in PDOs after being treated for 96 h. **p < 0.01. J,K) Relative spheroid area in PDO‐8912 transfected with the control or *XBP1s*‐overexpressing plasmid following treatment with palbociclib and fulvestrant for the indicated days. The culture medium was replaced every 3 d. *p < 0.05, **p < 0.01, ***p < 0.001. For statistical analysis, two‐tailed unpaired Student's *t*‐test was utilized for (B), (F), (G), (H), (I), (J), and (K). Data are presented as the mean ± standard deviation.

To determine how XBP1s regulate resistance to endocrine therapy plus CDK4/6 inhibitors, we overexpressed XBP1s in MCF7 and T‐47D cells and examined the impact on cell sensitivity to the drugs. According to the IC_50_ (half‐maximal inhibitory concentration) values, we found that XBP1s overexpression significantly desensitized cells to palbociclib (**Figure** **3**A), which was confirmed by the colony formation assay (Figure 3B). As the mechanism of CDK4/6 inhibitors was to prevent Rb phosphorylation and subsequent release of E2F, we proceeded to investigate the expression of components comprising the Rb–E2F complex in the XBP1s overexpressed and control cells after 24 h of palbociclib treatment. Western blotting analysis demonstrated a consistent upregulation of E2F1 expression in cells that stably overexpressed XBP1s than in the control cells, regardless of the addition of palbociclib. Furthermore, both control and XBP1s‐overexpressed cells showed comparable decreases in phosphorylated Rb following palbociclib treatment (Figure 3C; Figure S3A, Supporting Information). Meanwhile, cell cycle analysis suggested that XBP1s promoted G1 exit and eliminated palbociclib‐mediated blockade of G1 to S phase transition (Figure 3D; Figure S3B, Supporting Information). Thus, these findings imply that XBP1s overexpression may facilitate the cell cycle transition from G1 to S phase by upregulating E2F1. Moreover, elevated expression of XBP1s has been validated in endocrine‐resistant HR+ breast cancer cells previously and has been linked to resistance to both selective ER modulators and selective ER degraders, represented by tamoxifen and fulvestrant.^[^ ^17^ ^]^ In particular, prior studies revealed that overexpression of XBP1s conferred resistance to fulvestrant by transactivation of nuclear receptor coactivator 3 and NF‐κB, both of which played pro‐survival roles in HR+ breast cancer.^[^ ^13^, ^18^ ^]^ Consistently, the IC_50_ values indicated that XBP1s conferred resistance to fulvestrant in MCF7 and T‐47D cells, as confirmed by the colony formation assay (Figure 3E–G). Together, these data suggest that XBP1s overexpression may potentially function as a resistance mechanism shared by CDK4/6 inhibitors and fulvestrant.

Patient‐derived organoid (PDO) models have proven reliable in recapitulating tumor heterogeneity and architecture in breast cancer, emerging as promising preclinical models for predicting therapeutic response.^[^ ^19^ ^]^ Thus, a total of 12 organoids derived from HR+/HER2− breast tumors were developed for drug sensitivity assay with palbociclib or fulvestrant (Figure S4A,B, Supporting Information). Based on the expression levels of XBP1s, PDOs were divided into the XBP1s^high^ and XBP1s^low^ groups using the median value of XBP1s as cut‐off (Figure S4C,D Supporting Information). XBP1s^high^ PDOs exhibited reduced sensitivity to both palbociclib and fulvestrant individually than XBP1s^low^ PDOs (Figure 3H,I). To determine whether XBP1s confers resistance to palbociclib and fulvestrant, we overexpressed XBP1s in selected HR+/HER2− PDOs and performed drug sensitivity assays (Figure S4E, Supporting Information). As expected, XBP1s‐overexpressed PDOs showed decreased efficacy of both palbociclib and fulvestrant, which was observed as smaller changes in the sizes of the PDOs and a significant right shift of the dose–response curves (Figure 3J,K; Figure S4F,G, Supporting Information). In summary, our findings suggest that increased expression of XBP1s could contribute to the resistance of HR+/HER2− breast cancer cells to palbociclib and fulvestrant.

### 2.3 XBP1 Inhibition Sensitized HR+/HER2− Breast Cancer Cells to Palbociclib and Fulvestrant

**Figure 4:**
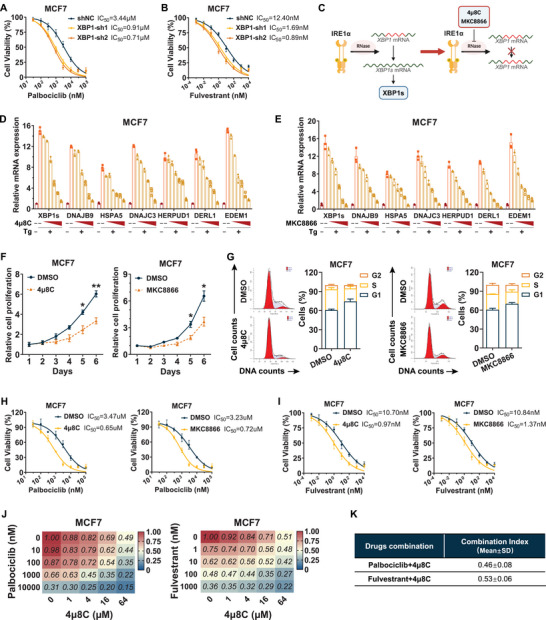
Epigenetic and pharmacological inhibition of *XBP1s* enhances the therapeutic sensitivity of fulvestrant and palbociclib in HR+/HER2− breast cancer cells. A) The dose–response curves and IC_50_ values of palbociclib in MCF7 cells with or without *XBP1* knockdown after being treated for 96 h. B) The dose–response curves and IC_50_ values of fulvestrant in MCF7 cells with or without XBP1 knockdown after being treated for 96 h. C) Schematic illustrating the mechanism through which 4µ8C and MKC8866 hinder the synthesis of XBP1s. D) The mRNA levels of *XBP1s* downstream target genes were assessed in MCF7 cells by the qRT‐PCR assay following 6 h of treatment with the vehicle control (dark red bars), 0.5 × 10^−6^ m of thapsigargin (Tg) alone (orange bars), or 0.5 × 10^−6^ m of Tg combined with increasing concentrations of 4µ8C (light yellow bars), from 1.25 to 40 × 10^−6^ m (twofold). E) The mRNA levels of *XBP1s* downstream target genes were assessed in MCF7 cells by the qRT‐PCR assay following 24 h of treatment with the vehicle control (dark red bars), 0.5 × 10^−6^ m of thapsigargin (Tg) alone (orange bars), or 0.5 × 10^−6^ m of Tg combined with increasing concentrations of MKC8866 (light yellow bars), from 0.125 to 4 × 10^−6^ m (twofold). F,G) Cell proliferation analysis and cell cycle distribution for MCF7 cells treated with or without 4µ8C and MKC8866. *p < 0.05, **p < 0.01. H) The dose–response curves and IC_50_ values of palbociclib in MCF7 cells transfected with or without 4µ8C (left) and MKC8866 (right) after being treated for 96 h. I) The dose–response curves and IC_50_ values of fulvestrant in MCF7 cells transfected with or without 4µ8C (left) and MKC8866 (right) after being treated for 96 h. J) Dose–response matrix (relative cell viability) of 4µ8C combined with palbociclib (left) or fulvestrant (right) in MCF7 cells based on the CCK8 assay. K) Combination index (CI) values for the antiproliferation effects of 4µ8C combined with palbociclib or fulvestrant in MCF7 cells. CI values were calculated based on the dose–response matrix in (J). For statistical analysis, two‐way ANOVA with Bonferroni's method correction was applied in (F). Data are presented as the mean ± standard deviation.

Next, we further explored whether epigenetic and pharmacological inhibition of *XBP1s* expression could improve the sensitivity of HR+/HER2− breast cancer cells to the treatment. First, both *XBP1* and *XBP1s* were stably knocked down in MCF7 and T‐47D cells by short hairpin RNA (shRNA) of XBP1 (Figure S5A,B, Supporting Information). Cell viability assays demonstrated a restored sensitivity to both palbociclib and fulvestrant in cells with *XBP1* knockdown (**Figure** **4**A,B; Figure S5C,D, Supporting Information). Selective IRE1α RNase inhibitors, 4µ8C and MKC8866, effectively blocked the splicing of *XBP1* mRNA and were employed to suppress XBP1s expression in vitro (Figure 4C).^[^ ^20^ ^]^ A substantial reduction in *XBP1s* mRNA level and its known downstream target genes was observed with escalating concentrations of 4µ8C and MKC8866 in thapsigargin‐treated MCF7 and T‐47D cells (Figure 4D,E; Figure S6A,B, Supporting Information). Consistently, XBP1s protein level also decreased with escalating concentrations of both inhibitors (Figure S6C, Supporting Information). Based on the IC_50_ value of 4µ8C and MKC8866 on inhibiting *XBP1s* mRNA level, MCF7 cells and T‐47D cells were treated with 7 × 10^−6^ m and 9 × 10^−6^ m of 4µ8C, and 0.15 × 10^−6^ m and 0.25 × 10^−6^ m of MKC8866, respectively (Figure S6D, Supporting Information). Cell viability and cell cycle assays showed that treatment with these two inhibitors decreased proliferation and led to G1 arrest in MCF7 and T‐47D cells (Figure 4F,G; Figure S6E,F, Supporting Information). IC_50_ assay further confirmed that drug efficacy was enhanced with the addition of 4µ8C and MKC8866 (Figure 4H,I; Figure S6G,H, Supporting Information). In addition, a pronounced synergistic effect in inhibiting cell proliferation was observed when 4µ8C was combined with palbociclib or fulvestrant in MCF7 and T‐47D cells (Figure 4J,K; Figure S6I,J, Supporting Information). Together, these results suggest that XBP1s could be a prospective target for enhancing the sensitivity of HR+/HER2− breast cancer cells to palbociclib and fulvestrant.

### 2.4 XBP1s Overexpression Promoted Cell Proliferation and G1/S Transition in HR+/HER2− Breast Cancer

**Figure 5:**
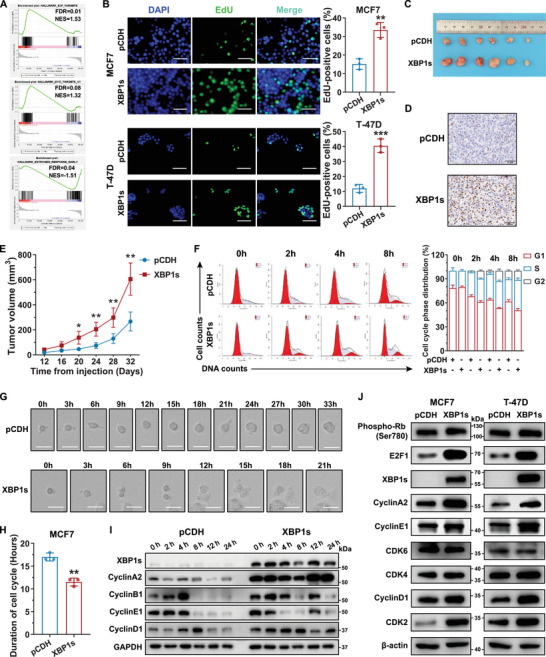
XBP1s overexpression promotes cell proliferation and cell cycle progression by regulating the G1/S transition in HR+/HER2− breast cancer cells. A) Enrichment plots for hallmarks of the E2F target, estrogen response, and the MYC target generated using gene set enrichment analysis (GSEA) based on the results of RNA sequencing of MCF7/pCDH and MCF7/XBP1s cells. FDR, false discovery rate. NES, normalized enrichment score. B) Representative images and quantification of EdU‐positive MCF7 and T‐47D cells with or without *XBP1s* overexpression. Scale bar, 50 µm. **p < 0.01, ***p < 0.001. C) Images of tumors from the MCF7/pCDH group and MCF7/XBP1s group at the end of the experiments. D) IHC staining for XBP1s in tumor slides from the MCF7/pCDH and MCF7/XBP1s groups. Scale bar, 50 µm. E) Tumor growth curves of cell‐derived xenografts of MCF7/pCDH and MCF7/XBP1s cells. Tumor volumes were recorded every 4 d starting from day 12 after the injection of cells. *n* = 6 per group. *p < 0.05, **p < 0.01. F–I) MCF7/pCDH and MCF7/XBP1s cells were synchronized to the G1/S phase by double thymidine blockage, then released to a fresh medium and collected for the indicated time. F) Cell cycle distribution was measured using flow cytometry. G) Representative time‐lapse images of MCF7 cells, with or without *XBP1s* overexpression, were presented at regular 3‐h intervals after synchronization. Scale bar, 25 µm. H) Quantification of the cell cycle duration based on time‐lapse live‐cell imaging. **p < 0.01. I) The levels of proteins related to cell cycle determination were detected by western blotting in MCF7 cells after synchronization and subsequent release for the indicated duration. J) Western blotting for the indicated proteins related to G1 to S phase transition in MCF7 and T‐47D cells with or without *XBP1s* overexpression. For statistical analysis, two‐tailed unpaired Student's *t*‐test was used for (B) and (H); two‐way ANOVA with Bonferroni's method correction was applied in (E). Data are presented as the mean ± standard deviation.

To explore the precise role of XBP1s in HR+/HER2− breast cancer, we performed RNA‐seq and GSEA to elucidate the transcriptional changes and predominant pathways related to XBP1s. Notably, the Myc targets, estrogen response early, and E2F targets were significantly enriched in both cell models, indicating that XBP1s may be positively correlated with cell proliferation and cell cycle promotion (**Figure** **5**A), which was confirmed by 5‐ethynyl‐2′‐deoxyuridine (EdU) and cell viability assays (Figure 5B; Figure S7A, Supporting Information). Consistent with these findings, XBP1s overexpression promoted tumor growth in the xenograft models (Figure 5C–E). In addition, flow cytometry revealed that *XBP1s*‐overexpressing MCF7 and T‐47D cells significantly accumulated in the S phase (Figure S7B, Supporting Information). After cell cycle synchronization, we confirmed that *XBP1s* overexpression led to accelerated entry into the S phase and reduced overall duration for cell doubling (Figure 5F–I). Given these results, we further detected the protein levels of markers involved in the G1/S transition to verify how *XBP1s* accelerate progression during the G1 to S phase. *XBP1s*‐overexpressed cells showed increased levels of several key regulatory proteins related to G1/S transition, including E2F1, cyclin A2, cyclin E1, and CDK2 (Figure 5J). Given that cyclin E and cyclin A2 are key downstream targets of E2F1,^[^ ^21^ ^]^ we speculate that XBP1s may enhance the G1/S transition by modulating E2F1 activity. Collectively, these findings highlight the significant role of XBP1s in cellular proliferation and cell cycle progression by controlling the G1/S transition.

### 2.5 XBP1s Transcriptionally Activated Staphylococcal Nuclease Domain‐Containing Protein 1 (SND1) in HR+/HER2− Breast Cancer

**Figure 6:**
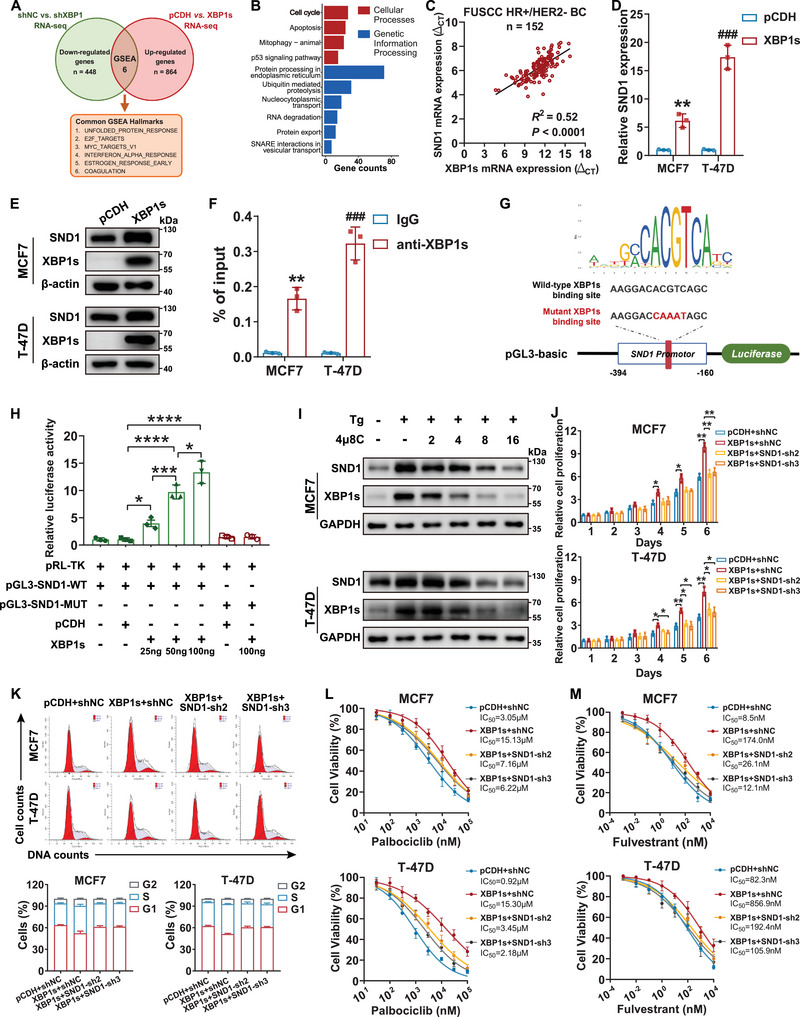
SND1 is a direct target of XBP1s and is responsible for the effects of XBP1s on cell proliferation, G1/S transition, and resistance to therapy. A) The Venn diagram shows the intersection of hallmark gene sets identified by GSEA based on the RNA‐seq data. B) KEGG pathway enrichment analysis of ChIP‐seq data obtained from MCF7 cells. C) Scatterplot showing the correlation between the expression of *XBP1s* and *SND1* in tumors obtained from patients with HR+/HER2− early breast cancer (*n* = 152). The correlation was assessed using Pearson's correlation coefficient. D,E) MCF7 and T‐47D cells were transfected with the control or *XBP1s*‐overexpressing plasmid. The mRNA expression of *SND1* was detected using qRT‐PCR assay (D), and the protein level of SND1 was measured via western blotting assay (E). **p < 0.01, ^###^ *p* < 0.001. F) ChIP‐qPCR of predicted XBP1s binding sites on *SND1* promoters in MCF7 and T‐47D cells. **p < 0.01, ^###^ *p* < 0.001. G) The upper panel shows the XBP1s binding motif; the lower panel shows a schematic diagram of dual‐luciferase reporter vectors containing wild‐type or mutant XBP1s binding sites on the SND1 promoter. H) The activity of wild‐type (pGL3–SND1–WT) and mutant (pGL3–SND1–MUT) SND1 promoter in MCF7 cells, as indicated, was measured by dual‐luciferase reporter assay. *p < 0.05, ***p < 0.001, ****p < 0.0001. I) The protein level of SND1 was detected using western blotting in MCF7 and T‐47D cells treated with the vehicle control, 0.5 × 10^−6^ m of Tg alone, or 0.5 × 10^−6^ m of Tg combined with increasing concentrations of 4µ8C. J–M) Cells were treated with shNC, SND1‐sh2, or SND1‐sh3 after being stably transfected with the control or *XBP1s*‐overexpressing plasmid. The cell proliferation analysis (J), cell cycle distribution (K), dose–response curves and IC_50_ values of palbociclib (L) and fulvestrant (M) in MCF7 and T‐47D cells. *p < 0.05, **p < 0.01. For statistical analysis, two‐tailed unpaired Student's *t*‐test was used for (D) and (F); one‐way ANOVA with Turkey's post hoc test was utilized for (H); two‐way ANOVA with Turkey's post hoc test was applied in (J). Data are presented as the mean ± standard deviation.

We conducted chromatin immunoprecipitation sequencing (ChIP‐seq) analysis to investigate the molecular mechanism by which XBP1s regulates G1/S transition. GSEA and Kyoto Encyclopedia of Genes and Genomes (KEGG) analyses were performed after integrating the results of ChIP‐seq with RNA‐seq, which indicated a common enrichment in cell cycle‐related pathways (**Figure** **6**A,B). Moreover, ChIP‐seq data demonstrated a significant enrichment of XBP1s binding site in the promoter region of SND1 (Figure S8, Supporting Information). SND1, a multifunctional conserved protein, is upregulated and associated with various types of cancer, including breast cancer.^[^ ^22^ ^]^ Furthermore, a positive correlation was observed between the expression of *XBP1s* and *SND1* in tumors obtained from patients with HR+/HER2− breast cancer (Figure 6C). Next, we confirmed that the expression of *SND1* mRNA and protein was significantly increased in *XBP1s*‐overexpressed MCF7 and T‐47D cells (Figure 6D,E). To validate the binding of XBP1s to the promoter region of SND1, we performed binding site prediction by JASPAR^[^ ^23^ ^]^ and identified a binding site with a relative score of 0.98 (Table S1, Supporting Information). ChIP‐qPCR assay showed significant enrichment of the predicted XBP1s binding site (Figure 6F). In addition, luciferase reporter plasmids containing the wild‐type sequence of the binding site on the SND1 promoter (pGL3–SND1–WT) or the corresponding mutated sequence (pGL3–SND1–MUT) were constructed to determine the direct binding of XBP1s to the promoter region of SND1 (Figure 6G). Dual‐luciferase reporter assay showed that overexpression of *XBP1s* significantly activated the promoter activity of SND1 in a dose‐dependent manner, but not the mutant, confirming that SND1 could be transcriptionally regulated by XBP1s (Figure 6H). Furthermore, the administration of 4µ8C resulted in a substantial decrease in the SND1protein level (Figure 6I). Taken together, these results revealed SND1 as a direct target of XBP1s.

### 2.6 SND1‐Driven Activation of E2F Target Genes May Mediate XBP1s‐Induced Proliferation and Drug Resistance

We further explored the functional roles of SND1 in HR+/HER2− breast cancer. Stable overexpression of *SND1* significantly enhanced the proliferative capacity and colony growth of MCF7 and T‐47D cells (Figure S9A–D, Supporting Information). In line with the functional role of XBP1s, SND1 promoted the acceleration of G1/S transition (Figure S9E, Supporting Information). Consistent with the latter, western blotting indicated increased protein levels of E2F1, cyclin A2, cyclin E1, and CDK2 in *SND1*‐overexpressing MCF7 and T‐47D cells (Figure S9F, Supporting Information). In contrast, *SND1* knockdown led to decreased expression levels of E2F1, cyclin A2, cyclin E1, and CDK2 (Figure S9G, Supporting Information). Moreover, we observed reduced aggressive phenotypes in *SND1* knockdown MCF7 and T‐47D cells, including suppressed cell proliferation, decreased colony formation capacity, and G1 arrest (Figure S9H–J, Supporting Information). Rescue experiments evaluating whether *SND1* suppression reversed the effects of XBP1s in HR+/HER2− breast cancer demonstrated that *SND1* knockdown partially impaired the XBP1s‐induced promotion of cell proliferation and G1/S transition (Figure 6J,K). In addition, *SND1* knockdown mostly re‐sensitized *XBP1s*‐overexpressed cells to palbociclib and fulvestrant (Figure 6L,M). Together, these results suggest that XBP1s‐induced tumor progression and drug resistance may be a consequence of the transcriptional activation of *SND1*.

**Figure 7:**
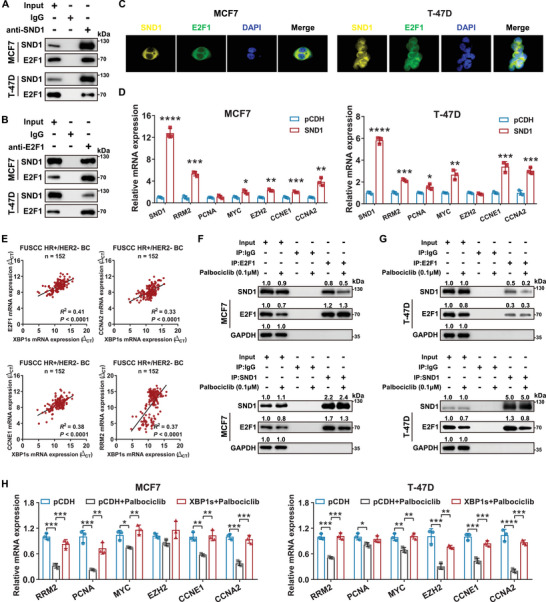
SND1 facilitates E2F1‐mediated transcriptional activation of target genes in HR+/HER2− breast cancer cells. A) Whole‐cell extracts of MCF7 and T‐47D cells were subjected to co‐immunoprecipitation (co‐IP) using anti‐SND1 antibodies, and the interaction between E2F1 and SND1 was identified via western blotting. B) Whole‐cell extracts of MCF7 and T‐47D cells were subjected to co‐IP using anti‐E2F1 antibodies, and the interaction between E2F1 and SND1 was identified by western blotting. C) Immunofluorescence (IF) staining assays exhibited the subcellular localization of SND1 (yellow) and E2F1 (green) in MCF7 and T‐47D cell lines. Nuclei were stained with DAPI (blue). D) The mRNA levels of *E2F1* downstream target genes in MCF7 and T‐47D cells after transfection with the control or *SND1*‐overexpressing plasmid were determined by qRT‐PCR assay. *p < 0.05, **p < 0.01, ***p < 0.001, ****p < 0.0001. E) Scatterplot showing the correlation between the expression of *XBP1s* and *E2F1*, *CCNE1*, *CCNA2*, and *RRM2* in tumors obtained from patients with HR+/HER2− early breast cancer (*n* = 152). The correlation was assessed using Pearson's correlation coefficient. F,G) After being treated with or without 0.1 × 10^−6^ of palbociclib for 24 h, whole‐cell extracts of MCF7 and T‐47D cells were immunoprecipitated with anti‐SND1 antibodies or anti‐E2F1 antibodies, followed by western blotting was conducted to investigate the interaction between SND1 and E2F1. H) The qRT‐PCR assay was used to detect the mRNA levels of *E2F1* downstream target genes in MCF7 and T‐47D cells treated with or without palbociclib following stable transfection with either the control or *XBP1s*‐overexpressing plasmid. *p < 0.05, **p < 0.01, ***p < 0.001, ****p < 0.0001. For statistical analysis, two‐tailed unpaired Student's *t*‐test was used for (D); one‐way ANOVA with Turkey's post hoc test was utilized for (H). Data are presented as the mean ± standard deviation.

SND1 has been identified as a transcriptional co‐activator of several transcription factors.^[^ ^22^ ^]^ In particular, previous studies have demonstrated that SND1 could promote the transcriptional activity of E2F1 and facilitate the transcriptional activation of E2F1 target genes, which play crucial roles in G1/S transition.^[^ ^24^ ^]^ In this regard, we confirmed the interaction between SND1 and E2F1 in breast cancer cells using a co‐immunoprecipitation (co‐IP) assay (**Figure** **7**A,B). We also demonstrated the co‐localization of SND1 and E2F1 by immunofluorescence (IF) analysis (Figure 7C). Meanwhile, the transcriptional activation of *E2F1* target genes upon *SND1* overexpression was verified using qRT‐PCR (Figure 7D). We also observed a positive correlation between the expression of *XBP1s* and *E2F1*, as well as *E2F1* targets in tumors obtained from patients with HR+/HER2− early breast cancer (Figure 7E). However, SND1 phosphorylation has been reported as a prerequisite for it to function as a co‐activator of E2F1, which could be induced by G1/S‐related CDKs, including CDK2, CDK4, and CDK6.^[^ ^24^ ^]^ Moreover, given that SND1 contributes to the degradation of a particular group of miRNAs that regulate the production of proteins involved in the G1/S transition, such as CDK2 and E2F1,^[^ ^25^ ^]^ we hypothesized that the inhibitory effects of CDK4/6 inhibitors on E2F1 targets expression might be counteracted by XBP1s‐induced transcriptional activation of SND1. Concordantly, we observed increased protein levels of E2F1 and CDK2 in *SND1*‐overexpressed MCF7 and T‐47D cells (Figure S9F, Supporting Information). Subsequently, we investigated whether CDK4/6 inhibitors can prevent SND1 from enhancing the transcriptional activation of E2F1 target genes under *XBP1s* amplification. Co‐IP assay revealed that the E2F1 protein level was reduced by palbociclib, whereas SND1 expression was unaffected. In addition, we noted a partial inhibition of the interaction between E2F1 and SND1 caused by palbociclib (Figure 7F,G). Furthermore, the qRT‐PCR assay showed that *XBP1s* amplification restored palbociclib‐induced suppression of the E2F1 targets (Figure 7H). Therefore, we speculated that CDK4/6 inhibitors can only partially block the interaction between SND1 and E2F1 and that this inhibitory effect can be reversed by XBP1s.

To further determine the effect of fulvestrant, palbociclib, or the combined therapy on the interaction between SND1 and E2F1 under XBP1s amplification in vivo, we detected the expression levels of SND1, E2F1, and the E2F1 target genes in MCF7 cell‐derived xenograft tumors (Figure 2). Tumors in the MCF7/XBP1s group exhibited elevated levels of SND1, E2F1, and Ki67 compared with tumors in the MCF7/pCDH group regardless of the treatment, while a comparable reduction in Rb phosphorylation levels was observed in both groups (Figure S10A–C, Supporting Information). E2F1 and Ki67 levels were significantly reduced in the MCF7/pCDH group after the administration of fulvestrant, palbociclib, and the combined therapy (Figure S10A,C, Supporting Information), whereas their levels remained stable in MCF7/XBP1s tumors upon treatment with fulvestrant or palbociclib individually, and showed a modest reduction with the combined therapy of palbociclib and fulvestrant (Figure S10B,C, Supporting Information). Furthermore, qRT‐PCR assay demonstrated that MCF7/XBP1s showed higher expression of E2F1 target genes across the treatment groups compared with MCF7/pCDH tumors (Figure S10D, Supporting Information).

Collectively, XBP1s enhances the cell cycle progression and confers drug resistance by activating the transcription of SND1, which could promote the upregulation of E2F1 target genes, thereby surpassing the blockade caused by CDK4/6 inhibitors.

### 2.7 4µ8C Sensitized HR+/HER2− Breast Cancer to the Combined Therapy of Palbociclib and Fulvestrant

**Figure 8:**
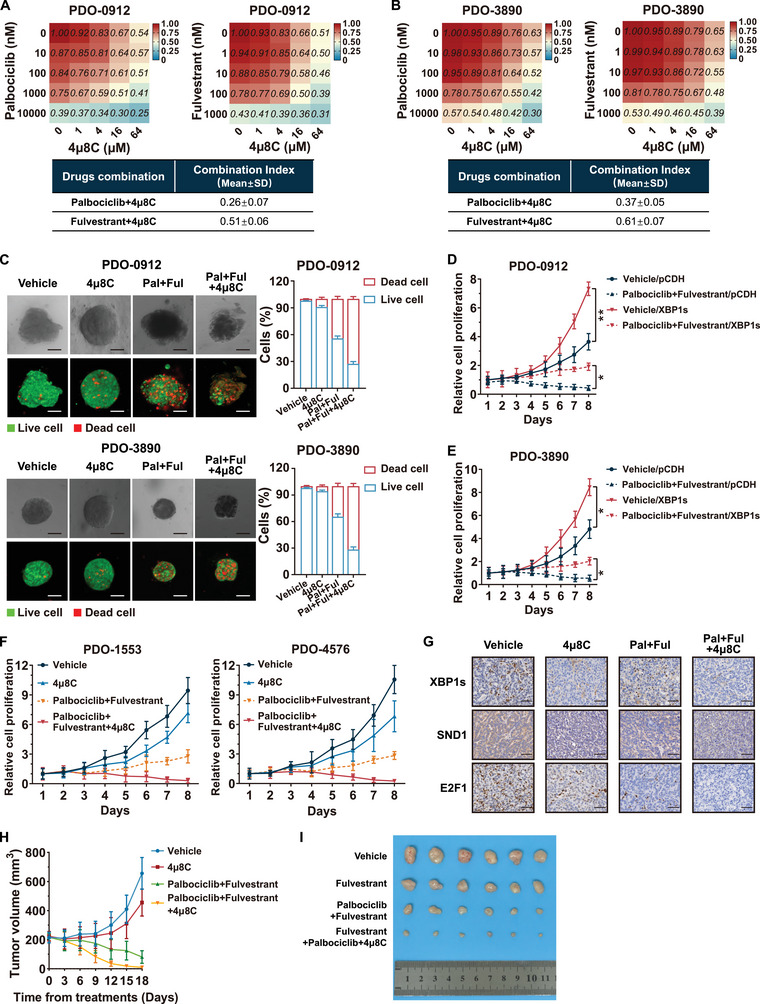
4µ8C sensitizes HR+/HER2− breast cancer to the combination therapy of fulvestrant and palbociclib in PDO models. A) Dose–response matrix (relative cell viability) and combination index (CI) for the effect of palbociclib (left) or fulvestrant (right) combined with 4µ8C in PDO‐0912. B) Dose–response matrix (relative cell viability) and CI for the effect of palbociclib (left) or fulvestrant (right) combined with 4µ8C in PDO‐3890. C) Acridine orange/propidium iodide (AO/PI) staining was performed on HR+/HER2− patient‐derived organoids PDO‐0912 and PDO‐3890 following treatment, as indicated. The left panel shows the representative images of live/dead organoids 96 h after the indicated treatment; the right panel shows the quantification results of live (green)/dead (red) analysis. PDO‐0912 was subjected to the vehicle, 10 × 10^−6^ m 4µ8C, a combination of 5 × 10^−6^ m palbociclib and 0.5 × 10^−6^ m fulvestrant (Pal+Ful), or their combination with 10 × 10^−6^ m 4µ8C (Pal+Ful+4µ8C); PDO‐3890 was subjected to the vehicle, 10 × 10^−6^ m 4µ8C, a combination of 14 × 10^−6^ m palbociclib and 1 × 10^−6^ m fulvestrant (Pal+Ful), or their combination with 10 × 10^−6^ m 4µ8C (Pal+Ful+4µ8C). Scale bar, 50 µm. D,E) Following transfection with the control or *XBP1s*‐overexpressing plasmid, cell viability assay was performed to determine the organoid growth following treatment with the vehicle and Pal+Ful for the indicated days in PDO‐0912 and PDO‐3890. *p < 0.05, **p < 0.01. F) Cell viability assay was performed to determine the organoid growth following treatment with the vehicle, 4µ8C, Pal+Ful, or Pal+Ful+4µ8C for the indicated days in PDO‐1553 and PDO‐4576. G–I) Mice with MCF7 cell‐derived xenografts were randomly divided into four treatment groups: vehicle, 4µ8C, Pal+Ful, or Pal+Ful+4µ8C. G) Representative images of IHC staining for XBP1s, SND1, and E2F1 in MCF7 cell‐derived xenografts from different treatment groups. Scale bar, 50 µm. H) Tumor growth curve plots of the MCF7 tumor in each treatment group after treatment administration. *n* = 6 per treatment group. I) Images of MCF7 cell‐derived xenografts at the end of the experiment. *n* = 6 per treatment group. For statistical analysis, two‐way ANOVA with Bonferroni's method correction was applied in (D) and (E). Data are presented as the mean ± standard deviation.

To confirm the crucial role of XBP1s in mediating resistance to CDK4/6 inhibitors and endocrine therapy, we assessed the responsiveness of HR+/HER2− breast cancer to the combined therapy of palbociclib and fulvestrant, with or without 4µ8C‐mediated blockade of *XBP1* splicing, in PDO‐0912 and PDO‐3890 models. As expected, *XBP1s* mRNA level was significantly downregulated after the administration of 4µ8C in both PDO models (Figure S11A, Supporting Information). In addition, the levels of downstream targets of XBP1s increased after thapsigargin treatment and consistently decreased with increasing concentrations of 4µ8C (Figure S11B,C, Supporting Information). Next, we evaluated the synergistic effect of palbociclib or fulvestrant in combination with 4µ8C in suppressing the development of PDOs. The combination index (CI) model validated the strong synergistic effect of 4µ8C with palbociclib and fulvestrant (**Figure** **8**A,B). Based on the IC_50_ values of fulvestrant and palbociclib in PDO‐0912 and PDO‐3890, we performed live/dead assay and observed a higher proportion of dead cells when treated with the combination treatment of palbociclib, fulvestrant, and 4µ8C than with palbociclib plus fulvestrant (Figure 8C; Figure S11D,E, Supporting Information). We also found lower levels of *SND1*, *E2F1*, and E2F1 target genes with the addition of 4µ8C (Figure S11F,G, Supporting Information). Consistent with our findings in the cell experiments, XBP1s significantly promoted cell growth and restored the proliferation of PDOs following treatment with the combined therapy of palbociclib and fulvestrant (Figure 8D,E). The cell viability and live/dead assays revealed that 4µ8C could enhance the therapeutic efficacy of palbociclib and fulvestrant in PDOs with high levels of XBP1s expression (Figure 8F; Figure S11H,I, Supporting Information). Moreover, to confirm the therapeutic efficacy of 4µ8C in vivo, we add the 4µ8C to the combined therapy of fulvestrant and palbociclib in the treatment of MCF7 xenografts. The administration of 4µ8C led to a substantial reduction of XBP1s expression and the SND1‐E2F1 axis (Figure 8G; Figure S11J, Supporting Information). The triple combination of 4µ8C, palbociclib, and fulvestrant showed favorable tolerability (Figure S11K, Supporting Information). Notably, the triple combination significantly suppressed the tumor growth compared with the double combination of palbociclib and fulvestrant (Figure 8H,I).

In conclusion, our findings suggest that XBP1s might act as a shared resistance mechanism for CDK4/6 inhibitors and fulvestrant, presenting an exploitable therapeutic vulnerability and prospective target for patients with HR+/HER2− MBC.

## 3 Discussion

The combination of CDK4/6 inhibitors and endocrine therapy substantially improves the survival outcomes of patients with advanced HR+/HER2− breast cancer, the emergence of drug resistance limits their clinical efficacy.^[^ ^26^ ^]^ Therefore, identifying the biomarkers for predicting efficacy and exploring the molecular basis of resistance to the combination of CDK4/6 inhibitors plus endocrine therapy is crucial for screening patients who may not benefit from the therapy and providing more effective alternative treatments.

In this study, we demonstrated *XBP1s* overexpression as a resistance mechanism shared by palbociclib and fulvestrant and associated with poor PFS in patients with HR+/HER2− MBC who received the combined therapy. Epigenetic or pharmacological suppression of *XBP1s* levels reversed its functions in facilitating G1/S transition and cell proliferation and restored cells’ responsiveness to palbociclib and fulvestrant. Based on in vivo and in vitro studies, we revealed that XBP1s enhanced the expression of E2F1 target genes by transcriptional activation of SND1, thereby exerting a pro‐survival effect in patients with HR+/HER2− breast cancer receiving the combined therapy. Hence, our study provides compelling evidence that XBP1s plays a crucial role in the progression of HR+/HER2− breast cancer and contributes to the development of resistance to the combined treatment of CDK4/6 inhibitors plus endocrine therapy.

The extensive use of the combination treatment of CDK4/6 inhibitors plus endocrine therapy highlights the need to identify patient subgroups that might benefit from this therapy to mitigate unnecessary drug toxicities and relieve the financial burden for patients with metastatic disease.^[^ ^27^ ^]^ Considering the differences in genetic profiles between the primary tumor and metastatic disease and the evolution during disease progression, obtaining pretreatment metastatic tissue biopsies is essential for investigating the determinants of the therapeutic response.^[^ ^6^, ^28^ ^]^ However, the acquisition of metastatic tissue samples remains a challenge owing to the nature and location of the metastatic disease, and the patients’ tolerability toward tissue biopsy. Therefore, despite significant research efforts, no single marker or cassette of markers has been approved in clinical practice to identify the eligible population for administering the combined therapy, except for the HR and HER2 status.^[^ ^29^ ^]^ Recent studies based on translational medicine and preclinical models have constructed the molecular landscape and introduced a new insight into the resistance mechanism to the drug combination in patients with HR+/HER2− MBC, paving the way for identifying predictive biomarkers and novel therapeutic targets. Wander et al. analyzed metastatic tumor samples from patients who received CDK4/6 inhibition via whole exome sequencing and reported that upregulation of AKT1, aurora A protein kinase, and cyclin E1 might potentially mediate resistance to CDK4/6 inhibitors and mediated significant resistance to fulvestrant in cell line models.^[^ ^30^ ^]^ Similar observations were reported in laboratory‐based studies.^[^ ^31^ ^]^ In addition, activation of the fibroblast growth factor receptor (FGFR) pathway, phosphatase and tensin homolog (PTEN) loss, and high p16 protein levels appeared to convey resistance to both CDK4/6 blockade and endocrine therapy.^[^ ^32^ ^]^ To our knowledge, our study is the first to indicate that elevated *XBP1s* expression may confer cross‐resistance to the combination treatment and elucidate its mechanism of action using transcriptomic analysis and functional experiments.

Emerging evidence suggests that high expression of *XBP1s* is involved in the oncogenesis, progression, and development of therapeutic resistance in breast cancer.^[^ ^12^, ^17^, ^33^ ^]^ Particularly, transcriptomic analysis and clinical‐based studies have revealed that elevated expression of *XBP1* is an important gene expression signature in estrogen‐positive breast cancer.^[^ ^34^ ^]^ In concordance, our data showed high expression of both *XBP1* and *XBP1s* specifically in HR+/HER2− breast cancer. XBP1s was reported to play a pro‐survival role and reduce the responsiveness to tamoxifen and fulvestrant through multiple mechanisms.^[^ ^17^, ^35^ ^]^ For instance, ectopic expression of *XBP1s* protects cells from antiestrogen‐induced apoptosis by upregulation of BCL2 and mitochondrial apoptotic pathways.^[^ ^36^ ^]^ XBP1s activates p65/RelA transcription, leading to the induction of NF‐κB, which functions as a driver for resistance to tamoxifen and fulvestrant.^[^ ^13^ ^]^ Consistent with previous reports, we confirmed that XBP1s reduced the therapeutic response of fulvestrant in HR+/HER2− breast cancer. In addition, we brought new insight into the mechanism of XBP1s‐mediated resistance to endocrine therapy, suggesting that XBP1s upregulation promoted cell proliferation and G1/S transition by activating SND1/E2F1. Moreover, our study reported a synergistic effect between fulvestrant and pharmacological inhibition of XBP1s in HR+/HER2− breast cancer cell lines, indicating that targeting XBP1s could be a valuable approach for the development of novel predictive and therapeutic strategies in fulvestrant‐resistant breast cancer.

Notably, high XBP1s expression was also associated with an impaired response to CDK4/6 inhibitors. CDK4/6 inhibitors could disrupt the CDK4/6–cyclin D‐mediated phosphorylation of Rb and initiation of the E2F pathway, resulting in G1 arrest in sensitive cells.^[^ ^4^, ^37^ ^]^ Nevertheless, given that the regulation of G1/S transition also involves cyclin E–CDK2 and other cell cycle‐unspecific signals, accumulating research has demonstrated that the efficiency of CDK4/6 inhibitors is limited owing to inherent or acquired resistance.^[^ ^38^ ^]^ Several oncogenic signaling pathways, such as the loss of FAT, and activation of the FGFR and PI3K–AKT–mTOR pathways, may drive cells to lose their dependence on the CDK4/6–cyclin D–Rb pathway and escape from CDK4/6 inhibition.^[^ ^32^, ^39^ ^]^ Here, we demonstrated that XBP1s could facilitate S‐phase entry and increase the expression levels of E2F1 and E2F1 targets with palbociclib treatment. Consequently, we speculate that XBP1s may evade the effect of CDK4/6 inhibitors by regulating the E2F1 pathway. Previous studies demonstrated a negative correlation between XBP1s and E2F1 in melanoma and fibrosarcoma models^[^ ^40^ ^]^ This divergence possibly reflects cancer‐type‐specific adaptations, as Acosta‐Alveary et al. noted that the regulatory networks of XBP1s follow a cell type‐ and condition‐specific manner.^[^ ^41^ ^]^ Therefore, the molecular mechanism governing XBP1s‐E2F1 regulation in HR+/HER2− breast cancer requires further investigation.

Next, we discovered XBP1s‐mediated transcriptional activation of SND1, a multifunctional transcriptional co‐activator that was previously reported to contain potential motifs for XBP1s binding by bioinformatic analysis.^[^ ^42^ ^]^ To function as a co‐activator of E2F1, SND1 needs to be phosphorylated, which could be enhanced by CDK2, CDK4, or CDK6; thereby, CDK4/6 inhibitors could partially impede this interaction.^[^ ^24^ ^]^ Interestingly, SND1 was also reported to increase the expression of proteins critical for G1/S transition, including E2F1 and CDK2, through degrading specific upstream miRNAs.^[^ ^25^ ^]^ Therefore, XBP1s‐mediated upregulation of SND1 may increase the expression of E2F1 and CDK2, promote SND1 phosphorylation, and trigger the activation of E2F1 downstream targets, thereby activating cell cycle progression from G1 to S in HR+/HER2− breast cancer. We confirmed that SND1 interacted with E2F1 and upregulated the expression of E2F1 targets, leading to the activation of the G1/S transition. In addition, XBP1s overexpression reversed the palbociclib‐induced downregulation of E2F1 target genes and impaired the therapeutic efficiency of palbociclib in HR+/HER2− breast cancer. Notably, previous studies indicated that the MYC, known as a direct target gene of E2F1,^[^ ^43^ ^]^ could enhance the activity of IRE1α RNase, resulting in elevated XBP1s/XBP1u ratio, and directly activate the transcription of XBP1 by binding to its promoter region.^[^ ^33^, ^44^ ^]^ Hence, these results suggest that XBP1s may participate in a positive feedback loop with E2F1 and MYC in breast cancer, furthering cell proliferation and the cell cycle in breast cancer. Moreover, previous studies established connections between XBP1s and the cell cycle regulator CDC6,^[^ ^45^ ^]^ which supports the regulatory function of XBP1s in cell cycle progression and indicates a potential mechanism for XBP1s‐mediated CDK4/6 inhibitor resistance. Thus, our findings add context to the resistance mechanism of CDK4/6 blockade, as high XBP1s level would help bypass the requirement of the CDK4/6–cyclin D–Rb pathway for cell cycle progression.

In light of the well‐established role of XBP1s in oncogenic transformation, cancer progression, and the development of drug resistance in breast cancer, our study further prompted us to investigate the feasibility and therapeutic potential of targeting XBP1s in HR+/HER2− breast cancer. We found that patients with tumors exhibiting elevated XBP1s level experienced significantly poorer PFS when receiving the combination treatment with CDK4/6 inhibitors and endocrine therapy. Moreover, in vivo experiments supported that XBP1s promoted tumor growth and correlated with a reduced response to palbociclib or fulvestrant, both alone and in combination. Due to technique limitations, direct pharmacological inhibition of XBP1s is unavailable.^[^ ^20a^ ^]^ Most preclinical investigations utilized small‐molecule inhibitors that target the IRE1a RNase domain to block XBP1s production. In this study, we confirmed that 4µ8C and MKC8866, two highly selective IRE1α RNase inhibitors, significantly reduced XBP1s level and suppressed the cell proliferation and cell cycle progression in HR+/HER2− breast cancer. In addition, 4µ8C was observed to exert synergistic efficacy with palbociclib and fulvestrant in HR+/HER2− breast cancer cell lines. Given that PDOs have been recognized as promising models for preclinical experiments of novel anticancer compounds and precision medicine,^[^ ^46^ ^]^ we assessed the impact of elevated expression of XBP1s and the efficiency of 4µ8C in HR+/HER2− PDOs. Our findings demonstrated that the anti‐tumor effect of the combination therapy of fulvestrant and palbociclib was substantially attenuated by the overexpression of XBP1s. In contrast, the addition of 4µ8C in XBP1s^high^ PDOs could enhance drug sensitivity and produce a synergistic effect comparable to what was seen in HR+/HER2− cell lines. While pharmacological inhibition of IRE1α RNase effectively reduced XBP1s levels and sensitized cells to CDK4/6inhibitors plus endocrine therapy in this study, an inherent limitation exists. The RNase domain of IRE1α mediates not only XBP1 mRNA splicing but also triggers regulated IRE1α‐dependent decay (RIDD), which degrades specific mRNAs and miRNAs with context‐dependent roles in cellular stress adaptation.^[^ ^47^ ^]^ Consequently, inhibiting IRE1α RNase concurrently suppresses both XBP1 production and RIDD. This lack of target specificity may lead to unintended biological effects and compromise the therapeutic potential of IRE1α RNase inhibitors.^[^ ^17a^ ^]^ Hence, our findings highlight the critical need to identify specific inhibitors for XBP1s or employ advanced strategies such as PROTACs to achieve targeted degradation of XBP1s, thus avoiding the pleiotropic effects of IRE1α RNase inhibition. Future preclinical and clinical studies are necessary to validate the safety and efficacy of targeting XBP1s for therapeutic intervention in patients with HR+/HER2− MBC.

## 4 Conclusion 

In summary, our study revealed the stimulatory effects of XBP1s on cell proliferation, cell cycle progression from G1 to S phase, and the development of cross‐resistance to palbociclib and fulvestrant by modulating the SND1/E2F1 axis in HR+/HER2− breast cancer. Patients with tumors exhibiting elevated XBP1s level may not be suitable for the combined therapy of CDK4/6 inhibitors and endocrine treatment. Moreover, while pharmacological inhibition of XBP1s using upstream IRE1α RNase inhibitors could retain the drug sensitivity, further exploration is needed to identify more specific approaches for XBP1s blockade.

## 5 Experimental Section

### Patient Specimens

Tumor specimens were collected from patients with pathologically confirmed breast cancer at Fudan University Shanghai Cancer Center (FUSCC). For the validation cohort, tissue biopsies were performed on the metastatic disease or recurrent tumor lesion before the initiation of metastatic treatment for patients who were diagnosed with advanced HR+/HER2− breast cancer and subjected to the combination therapy of CDK4/6 inhibitors and endocrine therapy. All of the biopsy specimens were obtained under imaging or endoscopic guidance and were confirmed as MBC tissue by at least two pathologists. The demographic, clinical, pathological, and therapeutic information of patients in this validation cohort were retrospectively obtained through medical record review and are provided in Table S2 (Supporting Information). In this study, the particular combined therapy regimen was determined by medical oncologists and administered as the first‐line treatment following the diagnosis of metastatic breast cancer. Endocrine therapy includes selective estrogen receptor modulators and aromatase inhibitors, while CDK4/6 inhibitors comprise palbociclib and abemaciclib. For the early breast cancer cohort, a total of 274 patients who were diagnosed with early breast cancer and received surgery at FUSCC were included. Basic demographic and clinicopathological information for the cohort is shown in Table S3 (Supporting Information). Patients were divided into three groups according to HR (estrogen receptor and progesterone receptor) and HER2 status, which includes HR+/HER2− subtype, HER2+ subtype, and triple‐negative (HR−/HER2−) subtype. All patients signed written informed consent for using samples for research purposes before inclusion in the study, as approved by the ethics committee of FUSCC (Approval No. 050432‐4‐2108). All the tissue biopsy samples were frozen in liquid nitrogen until use and stored at the Tissue Bank of FUSCC. The study protocol conformed to the ethical guidelines of the 1975 Declaration of Helsinki and was determined according to the American Joint Committee on Cancer Classification Criteria.

PFS was calculated as the period from the co‐administration of CDK4/6 inhibitors and endocrine therapy to the date of disease progression in the advanced setting. The physician determined the disease progression based on radiological examination. According to previous clinical trials, clinical benefit was defined as PFS > 6 months after initiation of the combined therapy.^[^ ^15^, ^48^ ^]^

### RNA Isolation, RNA‐seq, and Bioinformatics Analyses

RNA from the cell lines and tissues was extracted with TRIzol reagent (15596026, Invitrogen) according to the manufacturer's protocol. The total RNA of each sample was quantified and qualified by Agilent 2100/2200 Bioanalyzer (Agilent Technologies), and NanoDrop (Thermo Fisher Scientific Inc.). 1 µg total RNA was used for library preparation using NEBNext Ultra Directional RNA Library Prep Kit (E7420, New England Biolabs). The RNA sequencing (RNA‐seq) was performed on an Illumina HiSeq 3000 as 2×150 paired‐end reads. All generated FASTQ reads were aligned to a human reference genome (GRCh38/hg38) via the software Hisat2 (V 2.0.1). The RNA‐seq data of breast cancer tissue and the corresponding clinicopathological information from The Cancer Genome Atlas (TCGA) and Molecular Taxonomy of Breast Cancer International Consortium (METABRIC) datasets were obtained from cBioPortal (https://www.cbioportal.org).

All gene expressions levels (counts or fragments per kilobase million) were processed with R packages. The differential expression genes (DEGs) analysis was performed with limma (V 3.40.6) between HR+/HER2− and non‐HR+/HER2− groups from the TCGA and METABRIC databases^[^ ^49^ ^]^; DESeq2 (V 1.40.1) was utilized for DEGs analysis between the treatment‐sensitive and the treatment‐resistant groups^[^ ^50^ ^]^; EdgeR (V 3.42.4) was applied for DEGs analysis between MCF7/pCDH and MCF7/XBP1s cells, and MCF7/shNC and MCF7/shXBP1 cells.^[^ ^51^ ^]^ To identify DEGs, Benjamini–Hochberg false discovery rate (FDR)‐adjusted *p*‐value of < 0.05 (the cut‐off for DEGs analysis between the treatment‐resistant and treatment‐sensitive group were set at 0.1) and |log_2_ (fold change)| > 1 were set.

The GSEA was performed using GSEA software (V 4.1.0). For GSEA, FDR values < 0.25 were defined as statistically significant. The KEGG pathway analysis was conducted by clusterProfiler (V 4.8.1), and visualized by ggplot2 (V 3.4.2) R packages.

### Cell Culture and Reagents

Breast cancer cell lines MCF7, T‐47D, as well as HEK293T cells, were obtained from the American Type Culture Collection (ATCC). All of the cell lines underwent routine single nucleotide polymorphisms (SNP) and short tandem repeat (STR) authentication. In addition, cell lines were routinely tested for Mycoplasma during the study. MCF7 were cultured in MEM (ATCC modification) (L540KJ, BasalMedia) + 10% fetal bovine serum (FBS, Biological Industries) + 5 mg mL^−1^ insulin. T‐47D and HEK293T cells were cultured in DMEM (L110KJ, BasalMedia) with 10% FBS. All of the corresponding culture mediums were supplemented with 1% penicillin‐streptomycin (S110JV, BasalMedia). Cell lines were maintained at 37 °C in a humidified atmosphere with 5% CO_2_.

Palbociclib (PD‐0332991) HCl (S1116, Selleck), Fulvestrant (S1191, Selleck), Thapsigargin (HY‐13433, MCE), Cremophor EL‐40 (HY‐Y1890, MedChemExpress), 4µ8C (412512, Sigma‐Aldrich), and MKC8866 (HY‐104040, MedChemExpress) were purchased and utilized following the manufacturer's instructions. Stock solutions were diluted and stored according to the manufacturer's protocols.

### Plasmid Construction and Cell Infection

To construct overexpression plasmids, the cDNAs of XBP1s and SND1 were cloned into the pCDH‐CMV (#72265, Addgene) vector. The shXBP1 and SND1 plasmids were constructed with the pLKO.1 puro (#8453, Addgene) vector. To produce lentiviruses, the target plasmids mentioned above and empty vectors were separately co‐transfected with the pMD2.G (#12259, Addgene) and psPAX2 (#12260, Addgene) lentiviral packaging plasmids into HEK293T cells. The transfection medium was changed after 10 h, and the conditioned medium containing lentivirus was collected 72 h after transfection. To establish the stable strains, supernatants of lentiviruses were applied to target cells with 8 µg mL^−1^ of polybrene (TR‐1003, Sigma‐Aldrich) and incubated for 12 h. Subsequently, fresh complete medium containing 3 µg mL^−1^ of puromycin (ant‐pr‐1, InvivoGen) was used for selection. Lipofectamine 3000 Reagent (L3000015, Invitrogen) was utilized for transient transfection according to the manufacturer's instructions. The sequences used for plasmid construction are shown in Table S4 (Supporting Information).

### Western Blotting

After incubation under basal conditions or indicated treatment, whole‐cell lysates were generated by T‐PER (78510, Thermo Scientific) containing protease and phosphatase inhibitors cocktail (B14001 and B15001, Selleck). Protein concentration was determined using the BCA Protein Assay kit (PC0020, Solarbio). Equal amounts of protein were loaded onto 8%–10% SDS‐PAGE and electrophoretically transferred to 0.22 × 10^−6^ m polyvinylidene difluoride (PVDF) membranes (ISEQ00010, Millipore). The membranes were incubated with indicated antibodies and determined using ECL Plus Reagent (32132, Thermo Fisher). The primary antibodies used were as follows: GAPDH (HRP‐60004, Proteintech), β‐actin (66009‐1‐Ig, Proteintech), XBP1u (25997‐1‐AP, Proteintech), XBP1s (27901, Cell Signaling Technology), IRE1α (3294, Cell Signaling Technology), Phospho‐IRE1α S724 (ab124945, Abcam), E2F1 (ab288369, Abcam), Rb (9309, Cell Signaling Technology), Phospho‐Rb S780 (8180, Cell Signaling Technology), CyclinA2 (67955, Cell Signaling Technology), CyclinE1 (20808, Cell Signaling Technology), CyclinB1 (12231, Cell Signaling Technology), CDK6 (3136, Cell Signaling Technology), CDK4 (12790, Cell Signaling Technology), CyclinD1 (2978, Cell Signaling Technology), CDK2 (2546, Cell Signaling Technology), SND1 (ab65078, Abcam). The secondary antibodies used were as follows: Anti‐rabbit IgG, HRP‐linked (7074, Cell Signaling Technology), and Anti‐mouse IgG, HRP‐linked (7076, Cell Signaling Technology).

### Quantitative Real‐Time Polymerase Chain Reaction

Total RNA was isolated using TRIzol reagent (15596026, Invitrogen) following the manufacturer's protocol. The reverse transcriptase reaction was carried out using a HiScript III 1st Strand cDNA Synthesis Kit (R312‐01, Vazyme) according to the manufacturer's protocol. Real‐time quantitative PCR was performed on QuantStudio 7 Flex Real‐time PCR System (Applied Biosystems) using the ChamQ SYBR qPCR Master Mix (Q311‐02, Vazyme). All primers were provided in Table S4 (Supporting Information). Relative mRNA levels were calculated using the comparative threshold cycle (*C*_T_) ∆∆*C*_T_ method. ACTB was used as the internal control.

### Cell Cycle Analysis

For cell cycle synchronization, a total of 5 × 10^5^ cells were cultured in 60 mm dishes to achieve ≈50% confluence. Next, the standard growth medium was replaced by a medium containing 2 × 10^−3^ m thymidine (T9250, Sigma Aldrich). After incubating for 16 h, cells were washed three times with PBS and re‐fed with a growth medium for 9 h. Then the cells were treated with medium with 2 × 10^−3^ m thymidine again. After another 16 h of incubation, cells were synchronized at the G1/S border by the double thymidine block. Subsequently, cells were cultured in the normal medium, or treated with medium containing the vehicle control, palbociclib, or 4µ8C for the indicated time. For cell cycle analysis, cells were collected and fixed in 70% ice‐cold EtOH overnight at −20 °C, and stained in 20 µg mL^−1^ of propidium iodide (P4864, Sigma) along with 200 µg mL^−1^ of RNase A (EN0531, Thermo Scientific). Cell cycle profiles were measured with the Accuri C6 flow cytometry system and analyzed with CytoExpert 2.3 software.

### Cell Viability Assay

For the cell proliferation assay, cells were seeded in 96‐well flat‐bottomed plates with a density of 2500 cells/well in 100 µL of culture medium, allowing cells to attach overnight. Each plate was incubated and tested at 37 °C and 5% CO_2_ for the indicated time. For drug sensitivity assays, 100 µL cell suspensions containing 5000 cells were added to each well of 96‐well flat‐bottomed plates overnight. Subsequently, the standard growth medium was replaced by a treatment medium, and cells were treated for the indicated duration with different concentrations of palbociclib, fulvestrant, or a combination of drugs. Cell viability of the above‐mentioned assays was measured using the Cell Counting Kit‐8 (CCK8) (CK04, Dojindo) according to the manufacturer's instructions. Briefly, 10 µL CCK8 solution was added to each well and incubated for 3 h. Absorbance values at 450 nm of each well were measured by a microplate reader SpectraMax M5 (Molecular Devices). Dose‐response curves were generated by GraphPad Prism 8.0. The half‐maximal inhibitory concentration (IC_50_) value was defined as the concentration of drug required for a 50% reduction in growth and determined by GraphPad Prism 8.0 using a sigmoidal regression model. Based on the Chou–Talalay method for drug combination, CompuSyn software (ComboSyn, Inc.) was set up and used to determine the effects of drug combinations.^[^ ^52^ ^]^ The CompuSyn software provides a quantitative measurement for combination effects using the CI. A CI value between 0 and 1 suggests synergism, a CI value of 1 indicates an additive impact, and a CI value of more than 1 represents antagonism. The final CI is determined by calculating the mean of the values of CI at the average effect (Fa) > 0.5, using the dose‐effect data obtained from the cell viability assay.

### Colony Formation Assay

For colony formation assays, 3 × 10^3^ cells were seeded into six‐well plates. The culture medium was refreshed by the standard medium every 3 d, or replaced by indicated medium with drugs on Day 2 and changed every 3 d. At the indicated endpoints, the cells were fixed with methanol, and stained with 0.1% crystal violet solution (C6158, Sigma Aldrich). The cell colonies were then counted and imaged.

### Organoid Culture

PDOs of breast cancer were developed and cultured using the methods previously described.^[^ ^19b^ ^]^ Briefly, the fresh tissues were minced and digested in the digestion buffer containing an enzyme mixture of 200 U mL^−1^ collagenase type III (LS004183, Worthington), and 100 U mL^−1^ hyaluronidase (H3506, Sigma‐Aldrich) for 2 h at 37 °C. After treatment with red cell lysis buffer, cells were passed through 100‐µm cell strainers (352360, Falcon), and suspended in 50 µL of basement membrane extract (BME; 3533‐010‐02, R&D System) in 24‐well plates. Once BME turned solidified, 500 µL of PDO growth medium was added and replaced every 3 d. For passaging organoids, the organoid harvesting solution (3700‐100‐01, R&D System) was used to melt BME, while TrypLE (12604021, Gibco) was used to digest organoids into single cells. For the cell proliferation assay of PDO, cells were counted and plated at a density of 1 × 10^5^ cells/well in black ultra‐low attachment 384‐well (3830, Corning) with 5% BME. After being cultured for the indicated time or receiving the specific treatment, cell viability was determined using the CellTiter‐Glo 3D Cell Viability Assay (G9683, Promega). The clinicopathological characteristics of PDOs used in this study are provided in Table S6 (Supporting Information). The components for the breast cancer organoid culture medium are provided in Table S5 (Supporting Information). Organoids were routinely tested for mycoplasma.

### 5‐Ethynyl‐2′‐deoxyuridine Assay

For the EdU assay, cells were cultured in a 48‐well plate, and processed with the Cell‐Light EdU Apollo 488 kit (C10310, Ribobio). Briefly, cells were incubated with 50 × 10^−6^ m EdU buffer at 37 °C for 2 h, and then treated with 4% paraformaldehyde and 0.1% Triton X‐100. Next, the cells were stained with EdU and Hoechst 33342 solution. Images were taken with a fluorescence microscope (Leica). The proliferation rate was calculated as the ratio of the number of EdU‐positive cells (green cells) to the total number of Hoechst 33342‐positive cells (blue cells).

### Immunohistochemical (IHC) Staining

The formalin‐fixed paraffin‐embedded (FFPE) tumor specimens from patients with metastatic breast cancer were obtained from the pathology department of FUSCC. Tumors from the xenograft model were fixed in 4% paraformaldehyde, while the breast organoids were embedded in 2% agarose gel before being fixed in 4% paraformaldehyde. After being dehydrated and embedded in paraffin, IHC was performed on 5‐µm‐thick sections. The sections were de‐paraffinized, rehydrated, and antigenic retrieval, followed by treatment with 3% peroxidase to inactivate endogenous peroxidase. Then, the slides were incubated with primary antibodies against XBP1u (25997‐1‐AP, Proteintech), XBP1s (27901, Cell Signaling Technology), ER (21244‐1‐AP, Proteintech), PR (25871‐1‐AP, Proteintech), HER2 (18299‐1‐AP, Proteintech), Ki67 (27309‐1‐AP, Proteintech), SND1 (ab65078, Abcam), Phospho‐Rb S780 (ab173289, Abcam), or E2F1 (ab288369, Abcam) in a humidified at 4 °C overnight. Subsequently, HRP‐conjugated secondary antibodies were applied, which were anti‐mouse secondary antibody (111‐035‐144, Jackson), and anti‐rabbit secondary antibody (111‐035‐046, Jackson). Finally, staining was performed with the DAB system and hematoxylin.

The quantification of cells exhibiting positive staining was performed by a pathologist who was blinded to the origins of the samples and therapeutic outcomes of patients. The protein expression of each specimen was evaluated and assigned a score based on the percentage of positive staining (ranged from 0 to 4, with 0 representing <5% positive staining, 1 representing 5%–25% positive staining, 2 representing 26%–50% positive staining, 3 representing 51%–75% positive staining, and 4 representing 76%–100% positive staining) and staining intensity (ranged from 0 to 3, with 0 for no staining, 1 for weak staining, 2 for moderate staining, and 3 for strong staining). Consequently, the determination of the overall immunoreactive score entailed the multiplication of the intensity score by the score denoting the percentage of positively stained cells, with values ranging from 0 to 12.

### Immunofluorescence staining

Cells were seeded on 24‐well flat‐bottomed plates with a density of 3 × 10^5^ cells/well. After being fixed with 4% paraformaldehyde, permeabilized by 0.1% Triton X‐100, and blocked by 10% goat serum, cells were incubated with primary antibodies. FFPE slides from HR+/HER2− PDOs were subjected to IF staining after deparaffinization and antigen retrieval. XBP1s (27901, Cell Signaling Technology), SND1 (ab65078, Abcam), E2F1 (ab288369, Abcam), EPCAM (2929, Cell Signaling Technology), Vimentin (5741, Cell Signaling Technology), and alpha‐smooth muscle Actin (ab7817, Abcam) were used. Then cells were incubated with secondary antibodies, which are as follows: Goat anti‐Rabbit IgG (H+L) Cross‐Adsorbed Alexa Fluor 488 (A11008, Invitrogen), Goat anti‐Mouse IgG (H+L) Highly Cross‐Adsorbed Alexa Fluor 647 (A21236, Invitrogen), and Goat anti‐Rabbit IgG (H+L) Highly Cross‐Adsorbed Alexa Fluor Plus 555 (A32732, Invitrogen). The slides were mounted with VECTASHIELD Mounting Media (H1200, Vector Laboratories), allowing for simultaneous nuclear staining with DAPI and anti‐fade protection. Images were acquired with a Leica confocal microscope.

### Live‐Cell Time‐Lapse Analysis

Cells were seeded in 24‐well flat‐bottomed plates with a density of 300 cells/well in a growth medium. The Lionheart LX automated microscope base configuration (BioTek Instruments, Inc.) was used for live‐cell imaging. Cells were held in a humidified incubator at 37 °C and 5% CO_2_. Image acquisition was controlled and analyzed by Gen5 3.0 software (BioTek Instruments, Inc.). Time‐lapse images were captured at 20× magnification every 1.5 h for 48 h. The lengths of the cell cycle were quantified for the duration of the live imaging period. After each cell division, only one daughter cell was analyzed in subsequent cell cycles.

### Chromatin Immunoprecipitation, ChIP Sequencing, and ChIP‐qPCR

For ChIP preparation, cells were washed with ice‐cold PBS and cross‐linked with 1% formaldehyde. The cross‐linking reaction was then quenched by 1.25 m glycine. Total cell lyses were collected with IP lysis buffer (50 × 10^−3^ m Tris‐HCl, pH 8.1, 10 × 10^−3^ m EDTA, 1% SDS) supplemented with protease and phosphatase inhibitors cocktail (B14001 and B15001, Selleck). The chromatin was sheared to an average size of 300–1000 base pairs fragments using a Bioruptor Sonicator (Diagenode). Immunoprecipitation was carried out using XBP1s (27901, Cell Signaling Technology) and normal mouse IgG (sc‐2025, Santa Cruz) antibodies at 4 °C overnight with rotation. Next, the immunocomplexes were incubated with 50 µL of Dynabeads magnetic beads (10004D, Invitrogen) for 6 h. The precipitated protein–DNA complexes were then reverse‐crosslinked at 65 °C, followed by DNA purification using ChIP DNA Clean & Concentrator (D5205, Zymo Research).

For ChIP‐seq, DNA samples of ChIP were used for library construction using NEBNext ChIP‐Seq Library Prep Master Mix Set for Illumina (E6240, New England Biolabs) according to the manufacturer's protocol. The libraries were loaded on an Illumina HiSeq instrument for sequencing. ChIP‐seq reads were aligned to a human reference genome (GRCh38/hg38) via the software Bowtie2 (V 2.2.6). The analysis and annotation of enriched ChIP regions were conducted with MACS2 (V 2.1.0) and EdgeR (V 3.4.6).^[^ ^53^ ^]^

To quantify the binding of endogenous XBP1s to the promoters of candidate genes, ChIP‐qPCR was performed on the products of ChIP with ChamQ SYBR qPCR Master Mix (Q311‐02, Vazyme). All primers used are described in Table S4 (Supporting Information).

### Luciferase Reporter Assay

The promoter region of the human SND1 gene was analyzed using the JASPAR database.^[^ ^23^ ^]^ The sequence of 100 to −2000 bp from the transcription initiation site of SND1 was scanned on the JASPAR website (https://jaspar.genereg.net/). The relative profile score threshold was set as 80%. The predicted binding site of XBP1s and the corresponding mutated sequence on the promoter region of SND1 were inserted into the pGL3–basic plasmid (E1751, Promega). HEK293T cells were plated in 48‐well plates at a density of 5 × 10^4^ cells/well. On the next day, cells were transiently transfected with pGL3–SND1–promoter plasmid and pRL–TK plasmid, or pGL3–basic control plasmid and pRL–TK plasmid, together with XBP1s plasmid or pCDH plasmid as control. At 48 h after transfection, the luciferase activity was measured with the dual‐luciferase reporter assay system (E1910, Promega). The firefly‐luciferase activity was normalized by the renilla‐luciferase activity.

### Co‐immunoprecipitation Analysis

Cells were cultured in standard growth medium or previously treated with palbociclib (0.1 × 10^−6^ m) for 24 h until lysed with IP lysis buffer (50 × 10^−3^ m Tris‐HCl, pH 8.1, 10 × 10^−3^ m EDTA, 1% SDS) supplemented with protease and phosphatase inhibitors cocktail (B14001 and B15001, Selleck). Protein concentration was determined using the BCA Protein Assay kit (PC0020, Solarbio). An equal amount of total protein was incubated with SND1 (ab65078, Abcam) or E2F1 (3742, Cell Signaling Technology) antibodies in a roller at 4 °C overnight. While normal rabbit IgG (3900, Cell Signaling Technology) was used as a negative control. Then, 25 µL of Dynabeads magnetic beads (10004D, Invitrogen) were added into each IP sample and incubated with rotation for 3 h. 5% of the total cell lysates were used as input. The bound proteins were denatured by heat (95 °C) with 2× SDS loading buffer for 10 min. The supernatants from denaturation were finally subjected to immunoblotting with the indicated antibodies.

### Live/Dead Staining

Acridine orange/propidium iodide (AO/PI) staining solution (CS2‐0106, Nexcelom) was used to quantify PDO death following treatment. The treatments included the vehicle, 4µ8C, palbociclib in combination with fulvestrant, or a combination of palbociclib and fulvestrant along with 4µ8C. After being incubated in the dark at 37 °C for 20 min with the staining solution in a humidified atmosphere with 5% CO_2_, the images of PDO were captured by Leica confocal microscope equipped with Leica application suite X (LAS X) software platform. Following stepping through a z‐series of PDO using stacks, the z‐series were projected onto a single plane for processing image analysis. ImageJ was used to quantify the total area of PDOs dyed with acridine orange (live cell) or propidium iodide (dead cell).

### In Vivo Experiments

The designs of animal studies and procedures were approved by the Institutional Animal Care Committee (IACUC) of the FUSCC (Approval No. FUSCC‐IACUC‐S2022‐0378). Ethical compliance with IACUC protocols and institute standards was maintained.

A 0.36‐mg 17β‐estradiol pellet (SE121, Innovative Research of America) was embedded subcutaneously in the lower dorsal region of each nude mouse one week before the cell injection.

For xenograft studies, 5 × 10^6^ of indicated cells were resuspended in PBS and mixed 2:1 by volume with Matrigel (BD Biosciences), and injected into the fourth mammary fat pad of female NOD/SCID (4–6 weeks old, Model Animal Research Center) mice (*n* = 6 per group). Tumor length and width were measured by caliper from Day 12 after cell injection. For the drug efficacy study, tumor length and width were monitored by a caliper every 4 d. The tumor volumes were calculated as width^2^ × length/2. The administration of drugs started when the tumor size reached an average volume of 200 mm^3^. Fulvestrant was administered subcutaneously at a dose of 50 mg kg^−1^ weekly. Palbociclib was administered orally 5 d week^−1^ at 75 mg kg^−1^. 4µ8C was dissolved in 15% Cremophor EL + 85% Saline solution, and administered intraperitoneally every day at 10 mg kg^−1^. The body weight of each mouse was recorded every 3 d after the administration of drugs.

For each tumor, the percent change in tumor volume was calculated as [(Vf − V0) / V0] ×100 for each tumor, where V0 = initial volume (at the beginning of treatment) and Vf = final volume (at the end of treatment). A minimum 50% reduction in tumor volume from baseline was defined as tumor regression (R). An increase in tumor volume of 35% or more was defined as progressive disease (PD). Responses between +35% and −50% were classified as stable disease (SD).^[^ ^54^ ^]^

### Statistical Analysis

All experiments in vitro were repeated independently at least twice with similar results. Data analyses were performed using GraphPad Prism 8.0. Statistical analysis was performed as described in the figure legends. *P* < 0.05 was considered statistically significant (****, ^####^ *p* < 0.0001; ***, ^###^ *p* < 0.001; **, ^##^ *p* < 0.01; *, ^#^ *P* < 0.05). The Spearman rank correlation test was used to analyze the relationship between mRNA levels of different targets. Differences between experimental groups were analyzed using unpaired Student's *t*‐test or ANOVA analysis. Error bars represent the mean ± standard deviation of three independent experiments. The median values of mRNA expression were utilized as a cut‐off point for determining the distribution of expression levels for the target gene. Survival analysis was conducted via Kaplan–Meier analysis and compared with the log‐rank test.

## Conflict of Interest

The authors declare no conflict of interest.

## Author Contributions

Y.T.S. and S.Y.L. contributed equally to this work. J.W., Y.Y.C., J.Y.X., and Y.T.S. conceived this project, designed the experiments, and supervised the full process. Y.T.S., S.Y.L., X.J.Z., W.R.C., M.X., M.C., H.Y.R., D.W.E.L., and L.Y.Z. conducted the experiments, as well as collected and analyzed the related data. S.Y.L. was responsible for the bioinformatics analysis. Y.T.S. and Y.Y.C. collected tissue and patient information. Y.T.S. and S.Y.L. prepared the figures and wrote the original draft of the manuscript. The order of the co‐first authors was determined according to their respective contributions and efforts in the study. All authors read and approved the final manuscript.

## Ethics Approval and Patient Consent Statement

All human samples were anonymized and de‐identified. Written informed consent for using samples for research purposes was signed by all of the patients before inclusion in the study, as approved by the ethics committee of Fudan University Shanghai Cancer Center (FUSCC) (Approval No. 050432‐4‐2108). All animal experiments were approved by the Institutional Animal Care Committee (IACUC) of the FUSCC (Approval No. FUSCC‐IACUC‐S2022‐0378).

## Supporting information



Supporting Information

## Data Availability

The raw sequencing data reported in this paper have been deposited in the Genome Sequence Archive in the National Genomics Data Center, China National Center for Bioinformation/Beijing Institute of Genomics, Chinese Academy of Sciences with BioProject number PRJCA033234.
