# Supplementary material for: XBP1s Mediates Cross‐resistance to Combination Treatment of CDK4/6 Inhibitors plus Endocrine Therapy in Breast Cancer
Source: Adv Sci (Weinh). 2025 Sep 12;12(44):e09588. doi: 10.1002/advs.202409588 (PMC12667467; doi:10.1002/advs.202409588)
Supplement: Supplementary file 1 — Supporting Information [file ADVS-12-e09588-s001.pdf]

XBP1s mediates cross-resistance to CDK4/6 inhibitors plus endocrine therapy in breast cancer.

Yuting Sang[1,2], Shiyang Liu[1,2], Xujie Zhou[1,2], Weiru Chi[1,2], Min Xiong[1,2], Ming Chen[1,2], Hengyu Ren[1,2], Douwaner Liu[1,2], Liyi Zhang[1,2], Jingyan Xue[1,2*], Yayun Chi[1,2*], Jiong Wu[1,2*]

[1] Department of Breast Surgery, Key Laboratory of Breast Cancer in Shanghai, Fudan University Shanghai Cancer Center, Shanghai, China.

[2] Department of Oncology, Shanghai Medical College, Fudan University, Shanghai, China.

Supporting Information

Supplementary Figures 1-11

Supplementary Tables 1-6

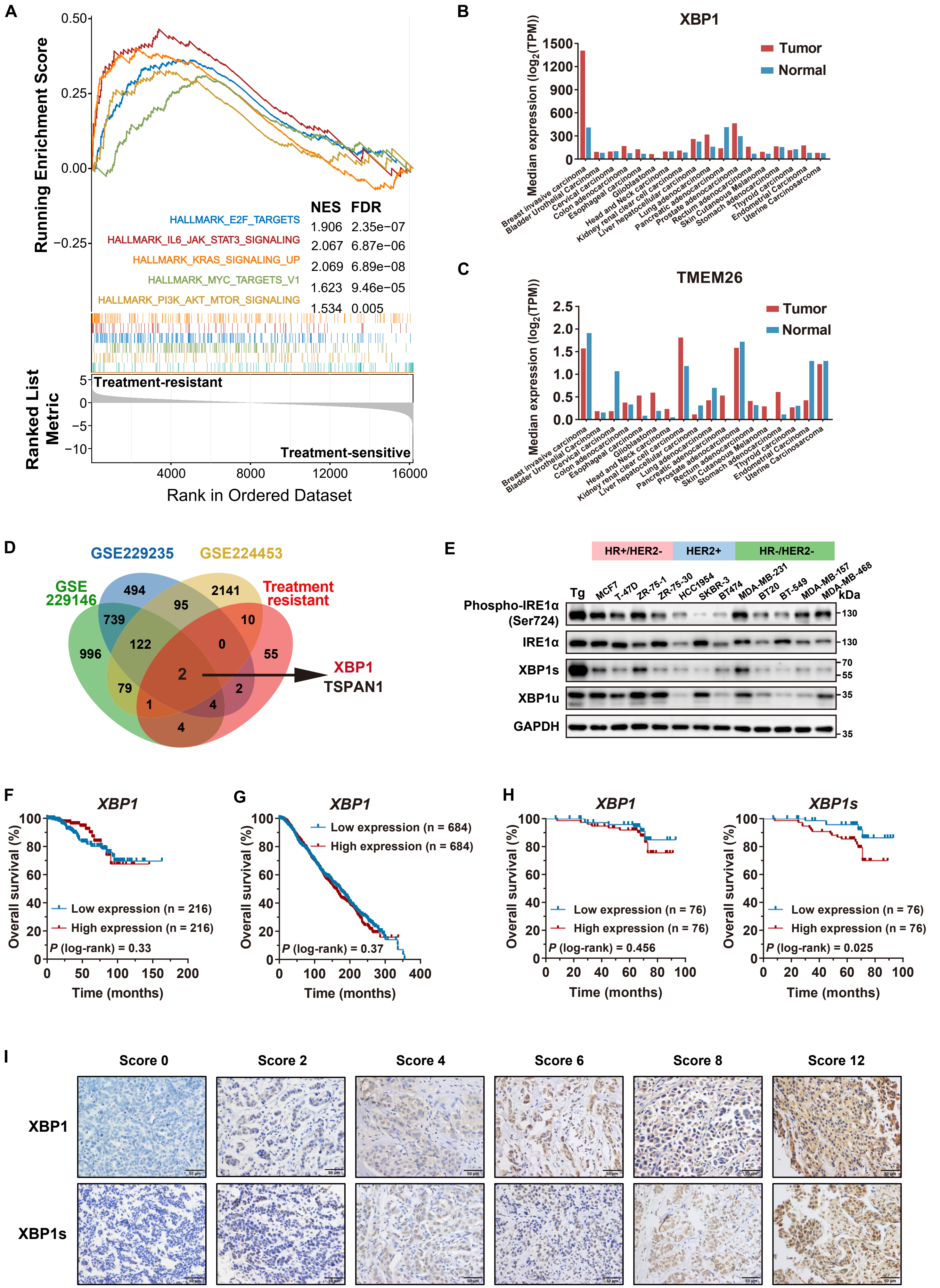

Supplementary Figure 1

Supplementary Figure 1. XBP1s is highly expressed in breast cancer and associated with less therapeutic response to CDK4/6 inhibitors and endocrine therapy in patients with HR+/HER2− metastatic breast cancer. (A) The signaling pathways that are enriched in the treatment-resistant cohort by gene set enrichment analysis (GSEA). FDR, false discovery rate. NES, normalized enrichment score. **(B)** The *XBP1* expression level across various solid tumor samples and paired normal tissues in the TCGA dataset. TPM, transcripts per million. **(C)** The *TMEM26* expression level across various solid tumor samples and paired normal tissues in the TCGA dataset. TPM, transcripts per million. **(D)** Venn diagram showing the shared numbers and overlaps of upregulated genes identified in GSE224435, GSE229146, GSE229235, and treatment-resistant cohorts. **(E)** Western blotting assay showed the protein levels related to the activation of the IRE1α-XBP1 pathway in different breast cancer cell lines. The cell lysate of MCF7 cells following treatment of 0.5 μM Thapsigargin (Tg) for 6 h was served as the positive control. **(F)** Kaplan–Meier curves of overall survival (OS) in the TCGA cohort of patients with high and low levels of *XBP1* expressions. **(G)** Kaplan–Meier curves of OS in the METABRIC cohort of patients with high and low levels of *XBP1* expressions. **(H)** Kaplan–Meier curves of OS in the patients with HR+/HER2− early breast cancer with high and low levels of *XBP1* and *XBP1s* expressions. **(I)** The representative images exhibit the respective levels of XBP1 and XBP1s expression, and indicated immunoreactive scores denoting the percentage of positive staining and staining intensity. Scale bar, 50 μm. *P*-values are indicated and were calculated using the log-rank test in **G-H**.

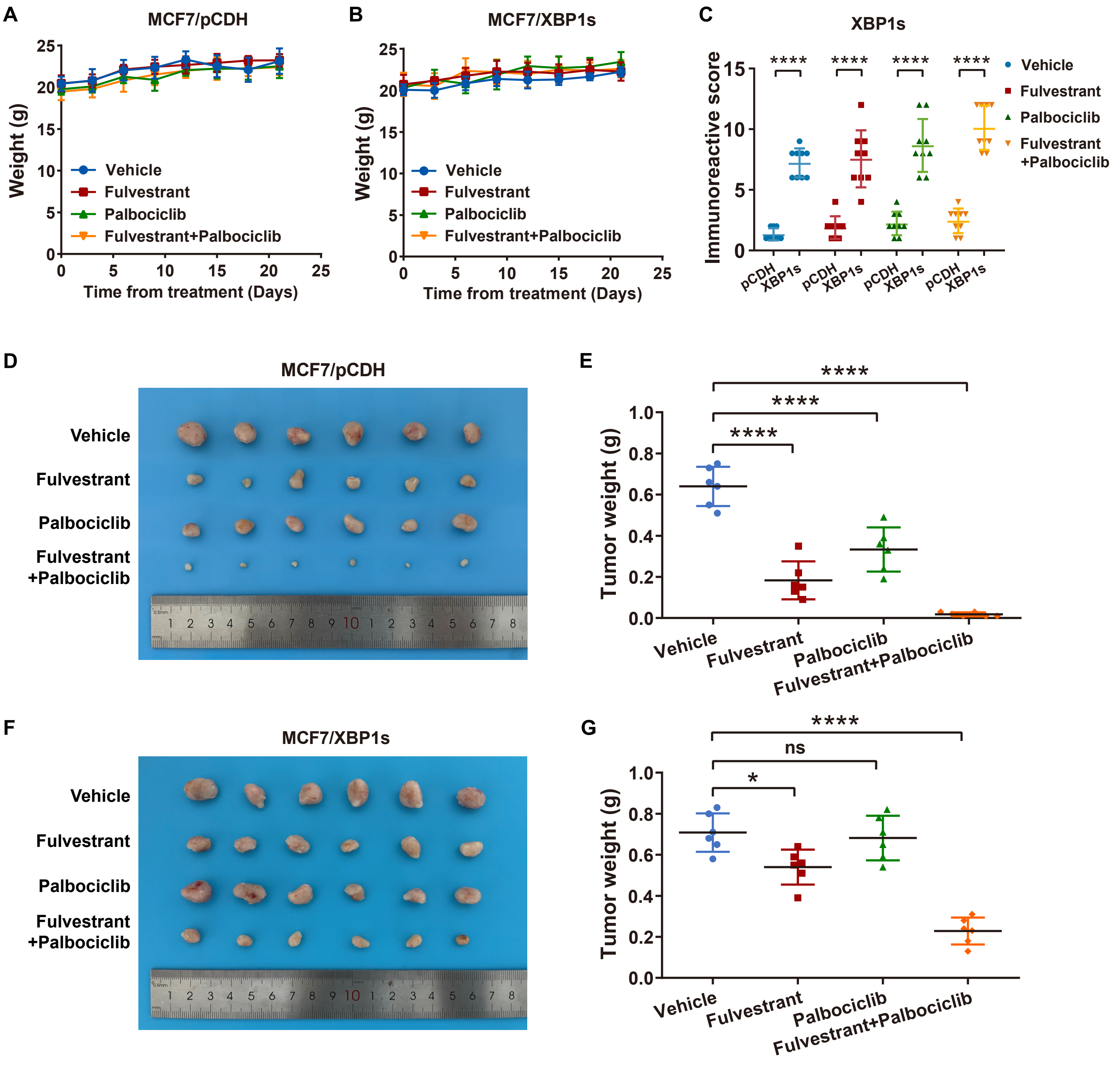

Supplementary Figure 2

Supplementary Figure 2. XBP1s diminishes the efficacy of the palbociclib and fulvestrant combination in suppressing tumor growth. (A and **B)** Changes in the body weight of mice bearing MCF7/pCDH **(A)** and MCF7/ XBP1s **(B)** cell-derived xenografts throughout the experiment, starting from day 0 of the treatment to the end (21 days after treatment). n = 6 per treatment group. **(C)** Quantification of XBP1s IHC staining from MCF7/pCDH and MCF7/XBP1s cell-derived xenografts in different treatment groups. n = 3 per treatment group; 3 fields per tumor. ****$P < 0.0001$. **(D** and **E)** Images **(D)** and tumor weights **(E)** of MCF7/pCDH cell-derived xenografts at the end of the experiment. n = 6 per treatment group. ****$P < 0.0001$. **(F** and **G)** Images **(F)** and tumor weights **(G)** of MCF7/XBP1s cell-derived xenografts at the end of the experiment. n = 6 per treatment group. ns, not significant; *$P < 0.05$, ****$P < 0.0001$. For statistical analysis, one-way ANOVA with Dunnett's post-hoc test was employed in **E** and **G**. Data are presented as the mean ± standard deviation.

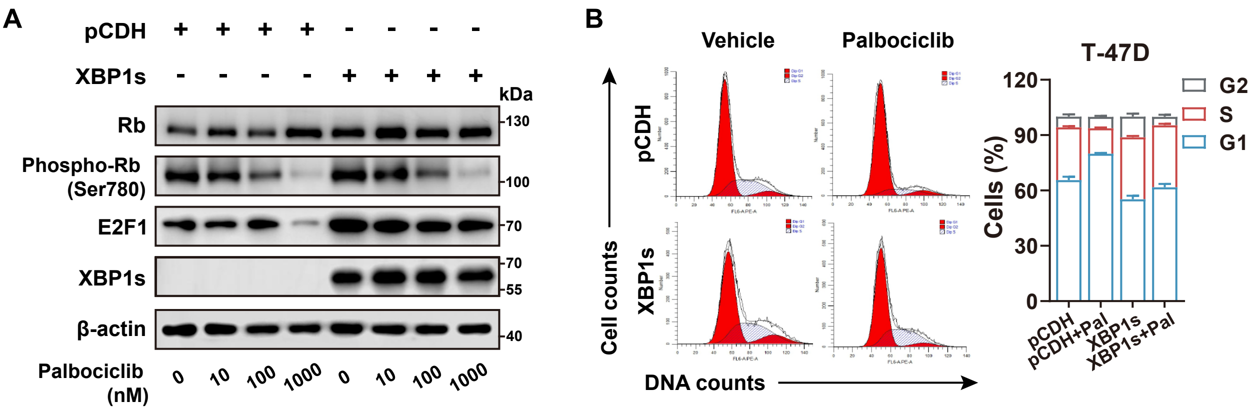

Supplementary Figure 3

Supplementary Figure 3. XBP1s impairs the blockade effect of palbociclib on the G1/S transition. **(A)** Western blotting of the indicated proteins in T-47D with or without *XBP1s* overexpression after being treated with specific concentrations of palbociclib. **(B)** Cell cycle distribution of T-47D cells with or without *XBP1s* overexpression after being treated with palbociclib. Data are presented as the mean ± standard deviation.

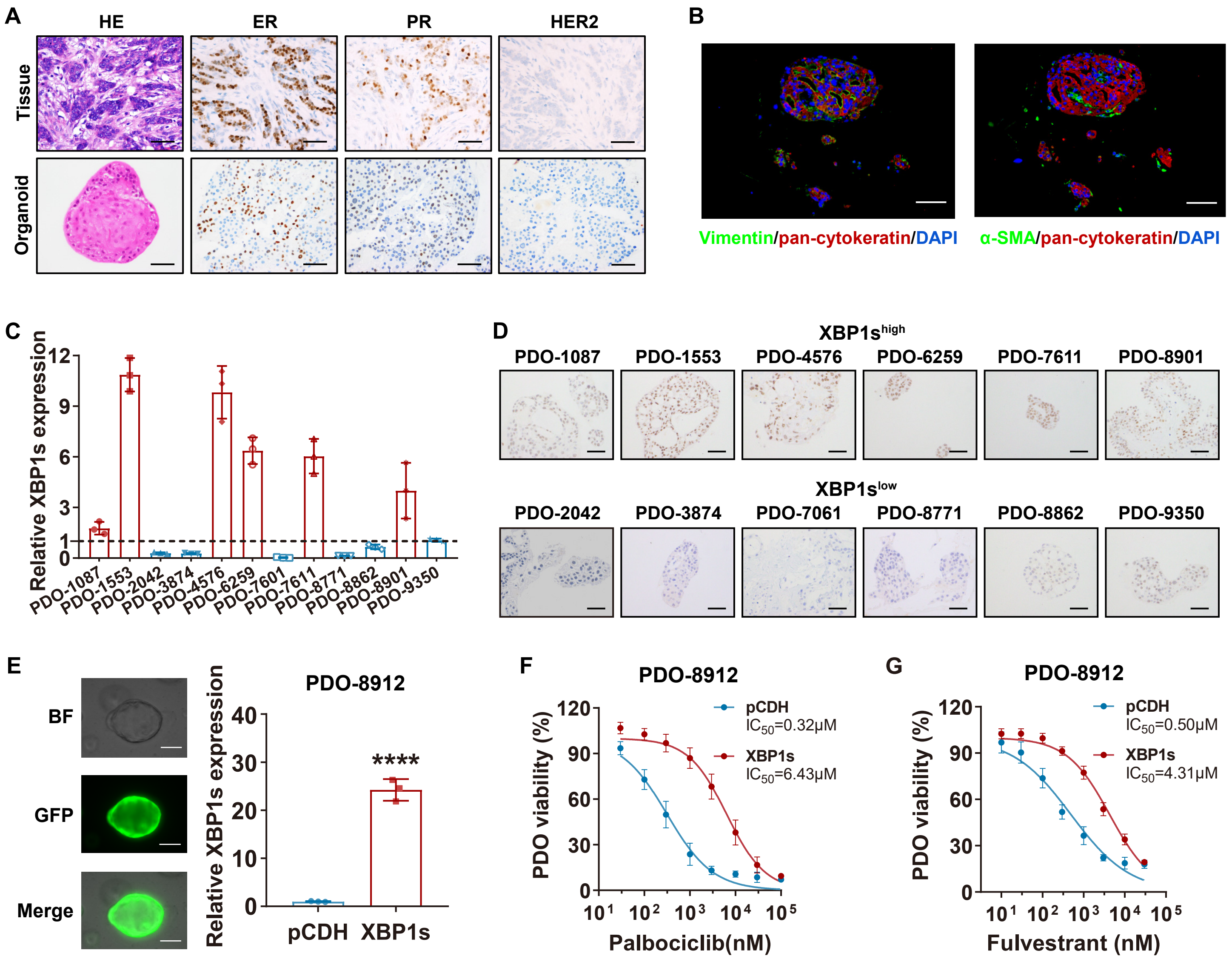

Supplementary Figure 4

Supplementary Figure 4. XBP1s reduces the therapeutic effect of fulvestrant and palbociclib treatment in the HR+/HER2− patient-derived organoid (PDO) models. (A) Representative histological sections of parental tumor tissue and HR+/HER2− PDOs models showed maintained expression of estrogen receptor (ER), progesterone receptor (PR), and human epidermal growth factor receptor 2 (HER2). Scale bar, 50 μm. **(B)** Immunofluorescence (IF) staining assays for different markers exhibiting cell components of PDO models. Nuclei were stained with DAPI. Scale bar, 50 μm. **(C)** *XBP1s* mRNA levels in 12 PDOs derived from HR+/HER2− breast tumors were detected by qRT–PCR assay. **(D)** Representative images of IHC staining for XBP1s in HR+/HER2− PDOs from the $XBP1s^{high}$ and $XBP1s^{low}$ groups. Scale bar, 50 μm. **(E)** Representative images (left) and *XBP1s* mRNA levels (right) of PDO-8912 after transfection with pCDH–GFP or XBP1s–GFP plasmid for 48 h. ****$P < 0.0001$. Scale bar, 50 μm. **(F** and **G)** The dose-response curves and half-maximal inhibitory concentration (IC_{50}) values of palbociclib **(F)** and fulvestrant **(G)** in PDO-8912 that were transfected with the control or *XBP1s*-overexpressing plasmid after being treated for 96 h. For statistical analysis, two-tailed unpaired student's *t*-test was used for **E**. Data are presented as the mean ± standard deviation.

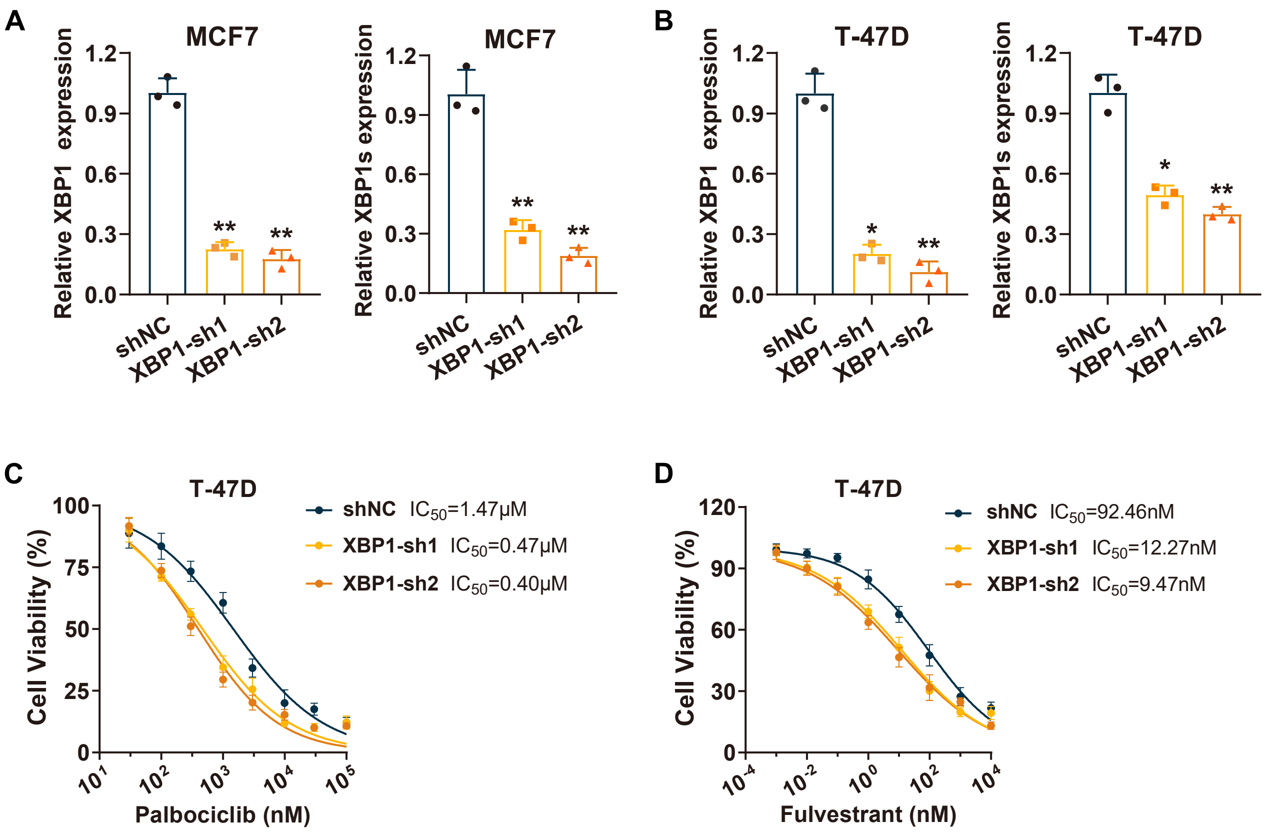

Supplementary Figure 5

Supplementary Figure 5. XBP1 knockdown results in increased sensitivity to palbociclib and fulvestrant in T-47D cells. (A and **B)** The XBP1 and XBP1s mRNA expression level in MCF7 **(A)** and T-47D **(B)** cells with or without *XBP1* knockdown were detected by qRT–PCR assay. $*P < 0.05$, $**P < 0.01$. **(C** and **D)** The dose-response curves and IC_{50} values of palbociclib **(C)** and fulvestrant **(D)** in T-47D cells with or without *XBP1* knockdown after being treated for 96 h. For statistical analysis, one-way ANOVA with Dunnett's post-hoc test was employed in **A** and **B**. Data are presented as the mean ± standard deviation.

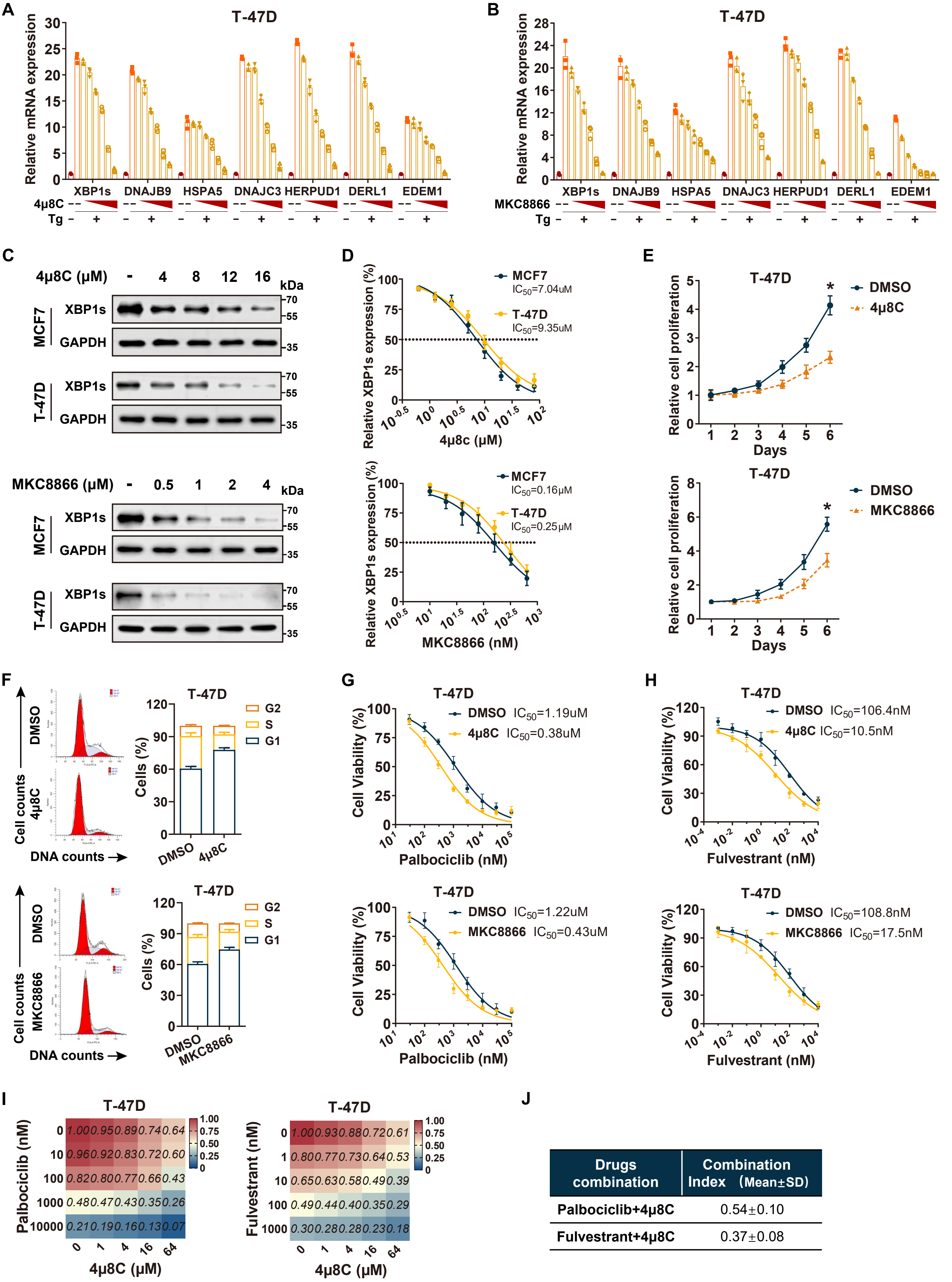

Drugs combination	Combination Index (Mean±SD)
Palbociclib+4μ8C	0.54±0.10
Fulvestrant+4μ8C	0.37±0.08

Supplementary Figure 6

Supplementary Figure 6. 4μ8C could effectively reduce the expression of XBP1s and show a synergistic effect with palbociclib and fulvestrant. (A) The mRNA levels of *XBP1s* downstream target genes were assessed in T-47D cells by qRT–PCR assay following 6 h of treatment with the vehicle control (dark red bars), 0.5 μM of thapsigargin (Tg) alone (orange bars), or 0.5 μM of Tg combined with increasing concentrations of 4μ8C (light yellow bars), from 1.25 to 40 μM (2-fold). **(B)** The mRNA levels of *XBP1s* downstream target genes were assessed in T-47D cells by qRT–PCR assay following 24 h of treatment with the vehicle control (dark red bars), 0.5 μM of thapsigargin (Tg) alone (orange bars), or 0.5 μM of Tg combined with increasing concentrations of MKC8866 (light yellow bars), from 0.125 to 4 μM (2-fold). **(C)** Western blotting of the indicated proteins in MCF7 and T-47D cells treated with increasing concentrations of 4μ8C or MKC8866. **(D)** The dose-response curves and IC_{50} values of 4μ8C and MKC8866 on inhibition of *XBP1* splicing in MCF7 and T-47D cells after being treated for 6 h. **(E** and **F)** The cell proliferation analysis **(E)** and cell cycle distribution **(F)** of T-47D cells treated with or without 4μ8C and MKC8866. *$P < 0.05$. **(G)** The dose-response curves and IC_{50} values of palbociclib in T-47D cells transfected with or without 4μ8C (left) and MKC8866 (right) after being treated for 96 h. **(H)** The dose-response curves and IC_{50} values of fulvestrant in T-47D cells transfected with or without 4μ8C (left) and MKC8866 (right) after being treated for 96 h. **(I)** Dose-response matrix (relative cell viability) of 4μ8C combined with palbociclib (left) or fulvestrant (right) in T-47D cells based on the CCK8 assay. **(J)** Combination index (CI) values for the anti-proliferation effects of 4μ8C combined with palbociclib or fulvestrant in MCF7 cells. CI values were calculated based on the Dose-response matrix in **(I)**. For statistical analysis, two-way ANOVA with Bonferroni's method correction was applied in **E**. Data are presented as the mean ± standard deviation.

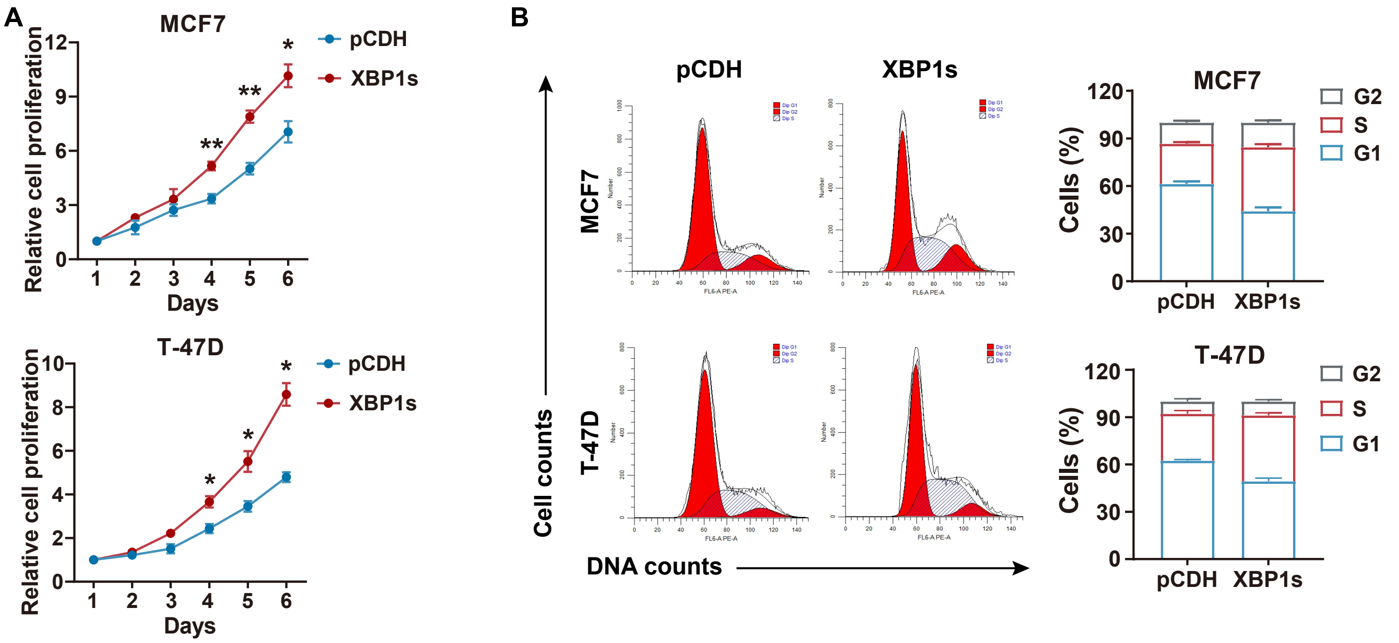

Supplementary Figure 7

Supplementary Figure 7. XBP1s promotes cell proliferation and G1/S transition in HR+/HER2− breast cancer cells. (A and **B)** MCF7 and T-47D cells were transfected with the control or *XBP1s*-overexpressing plasmid. CCK8 assay was performed to determine the cell viability **(A)**, and flow cytometry was utilized to analyze cell cycle distribution **(B)**. **P* < 0.05, ***P* < 0.01. For statistical analysis, two-way ANOVA with Bonferroni's method correction was applied in **A**. Data are presented as the mean ± standard deviation.

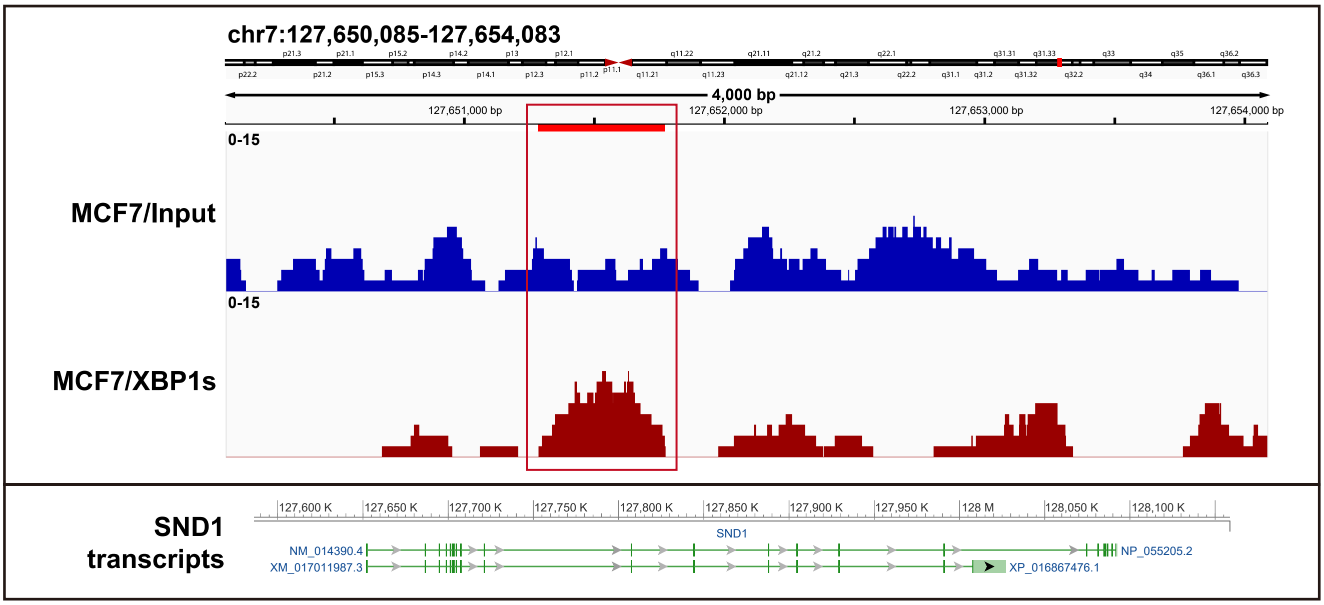

Supplementary Figure 8

Supplementary Figure 8. The ChIP-seq data demonstrated a significant enrichment of XBP1s binding site in the promoter region of SND1.

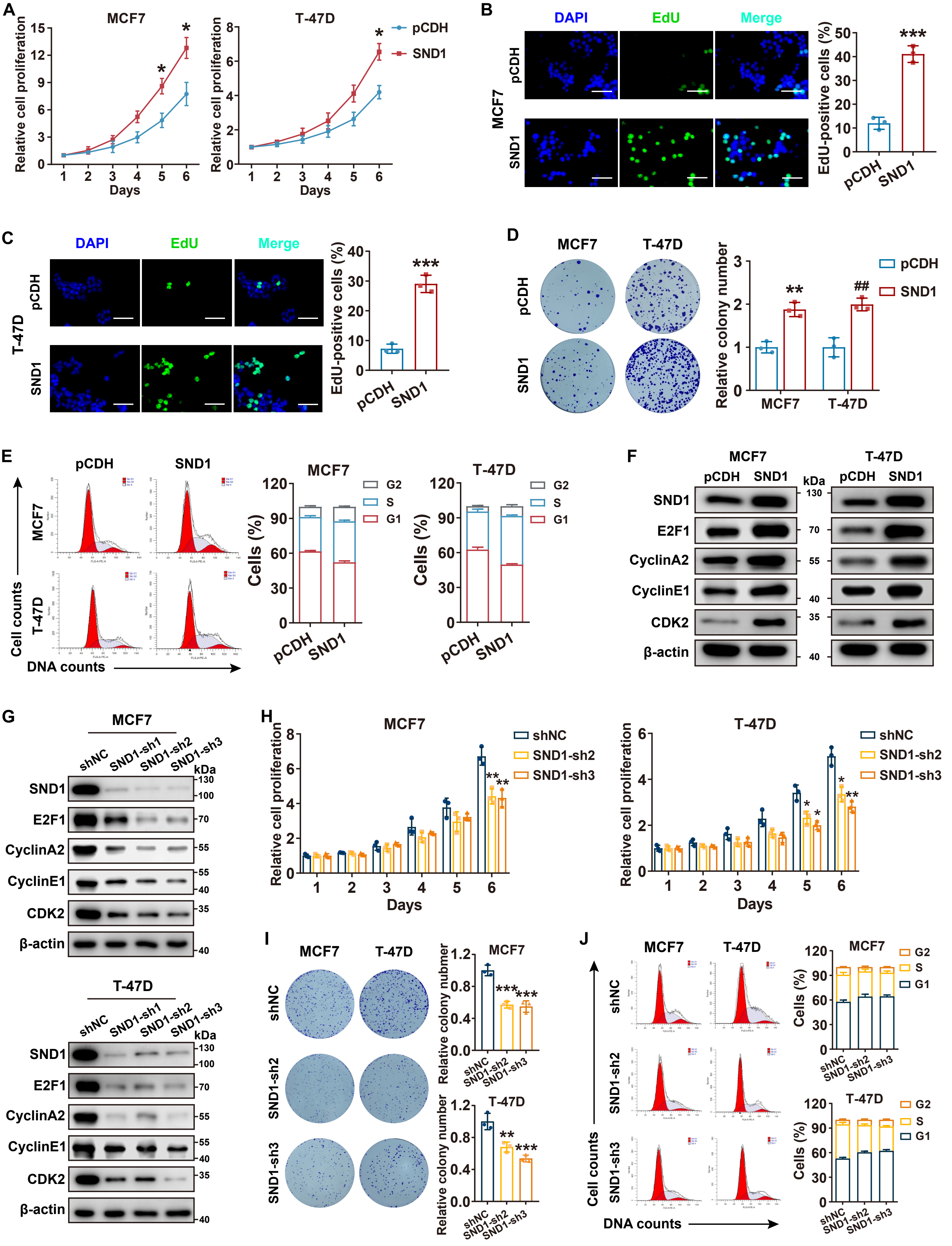

Supplementary Figure 9

Supplementary Figure 9. SND1 regulates cell proliferation and G1/S transition in HR+/HER2− breast cancer cells. (A–F) MCF7 and T-47D cells were transfected with the control or *SND1*-overexpressing plasmid. **(A)** CCK8 assay was performed to determine the cell viability. **P* < 0.05. **(B and C)** Representative images and quantification of EdU-positive MCF7 **(B)** and T-47D **(C)** cells. Scale bar, 50 μm. ****P* < 0.001. **(D)** Representative images and quantification of colony formation assay. **, ##*P* < 0.01. **(E)** The flow cytometry assay was utilized to analyze cell cycle distribution. **(F)** Western blotting assay showed the protein levels related to G1/S transition. **(G–J)** MCF7 and T-47D cells were transfected with shRNAs targeting *SND1* or control shRNAs. **(G)** Western blotting assay showed the protein levels related to G1/S transition. **(H)** CCK8 assay was performed to determine the cell viability. **P* < 0.05, ***P* < 0.01. **(I)** Representative images and quantification of colony formation assay. ***P* < 0.01, ****P* < 0.001. **(J)** Cell cycle distribution was detected by flow cytometry assay. For statistical analysis, two-tailed unpaired student's *t*-test was used for **B**, **C**, and **D**; two-way ANOVA with Bonferroni's method correction was applied in **A**; two-way ANOVA with Dunnett's post-hoc test was employed in **H**; one-way ANOVA with Dunnett's post-hoc test was utilized for **I**. Data are presented as the mean ± standard deviation.

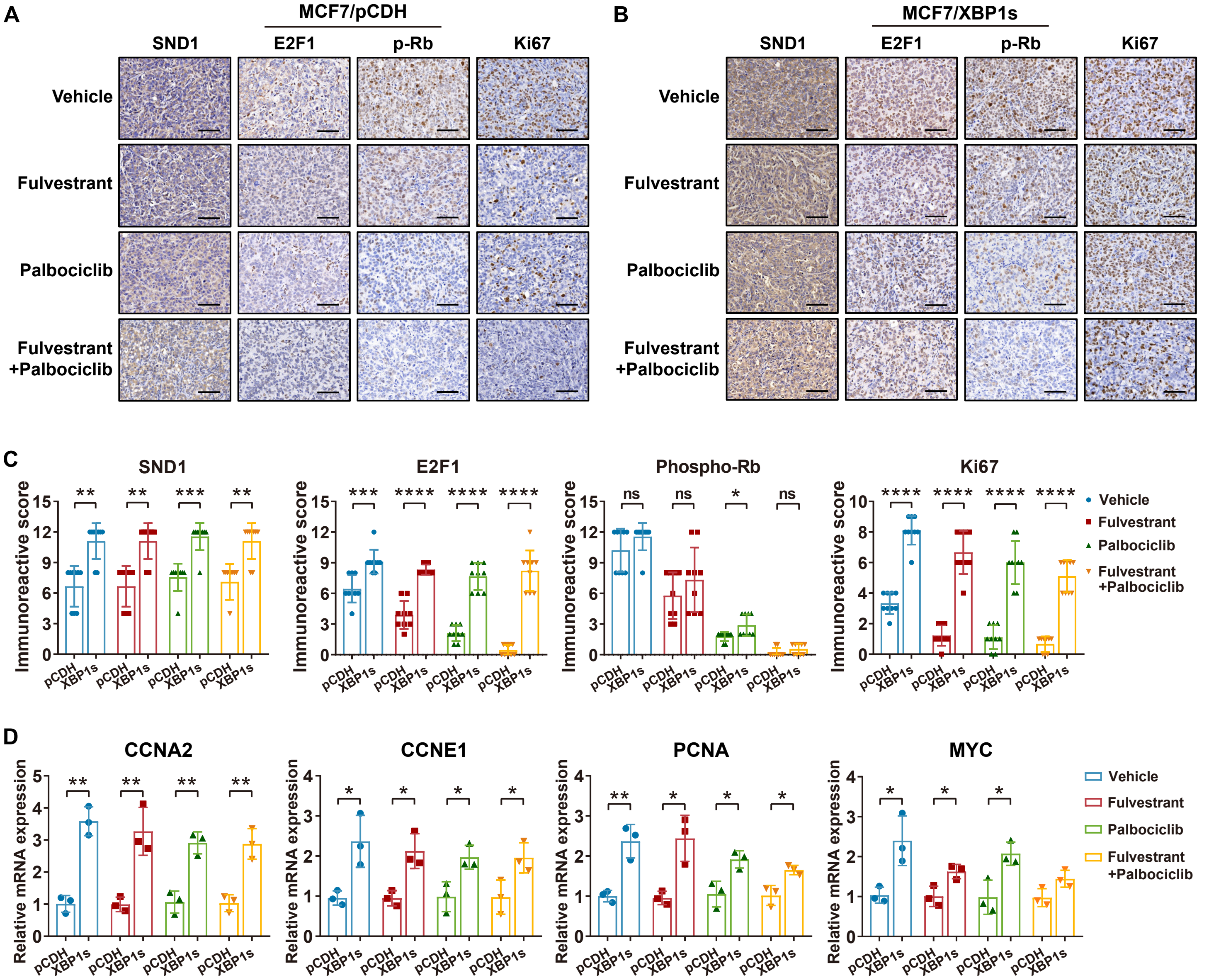

Supplementary Figure 10

Supplementary Figure 10. XBP1s could rescue the inhibitory effect of palbociclib and fulvestrant on the E2F1 pathway *in vivo*. **(A** and **B)** Representative images of IHC staining for SND1, E2F1, Ki67, and p-RB (phosphorylated Rb S780) in MCF7/pCDH **(A)** and MCF7/XBP1s **(B)** cell-derived xenografts from different treatment groups. Scale bar, 50 μm. **(C)** The immunoreactive scores of IHC staining for SND1, E2F1, phospho-Rb, and Ki67 in MCF7/pCDH and MCF7/XBP1s cell-derived xenografts from different treatment groups. n = 3 per treatment group; 3 fields per tumor. ns, not significant; *$P < 0.05$, **$P < 0.01$, ***$P < 0.001$, ****$P < 0.0001$. **(D)** The relative mRNA levels of E2F1 downstream target genes were detected by qRT–PCR assay in MCF7/pCDH and MCF7/XBP1s tumors from different treatment groups. Data are presented relative to the MCF7/pCDH tumor in the each treatment groups. n = 3 per treatment group. *$P < 0.05$, **$P < 0.01$. For statistical analysis, Mann–Whitney test was employed in **C**; two-tailed unpaired student's *t*-test was used for **D**. Data are presented as the mean ± standard deviation.

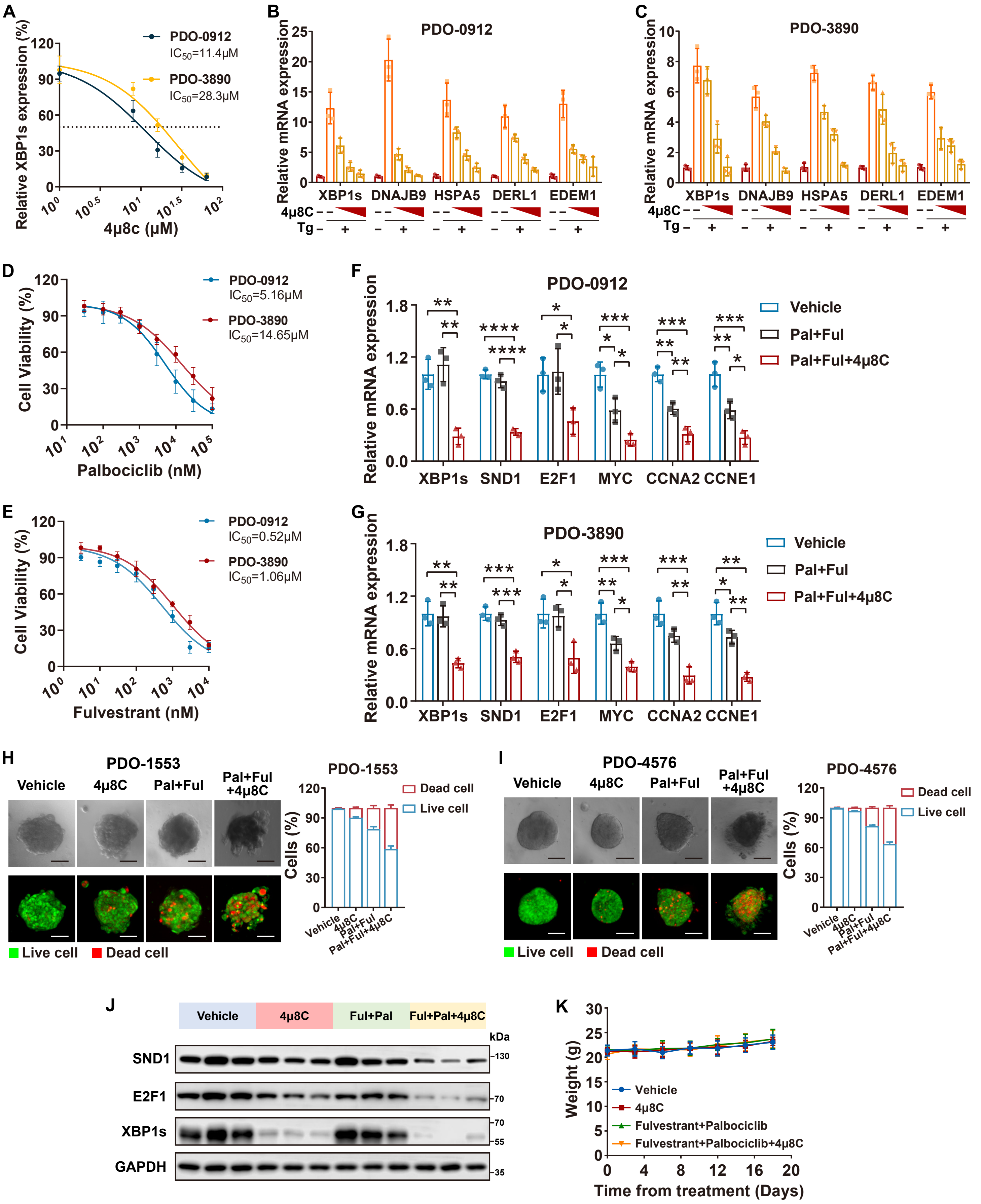

Supplementary Figure 11

Supplementary Figure 11. 4μ8C could effectively decrease the expression of XBP1s and downregulate the E2F1 targets in PDO models. (A) The dose-response curves and IC_{50} values of 4μ8C on inhibiting *XBP1* splicing in PDO-0912 and PDO-3890 after being treated for 6 h. **(B** and **C)** The mRNA levels of XBP1s downstream target genes were assessed in PDO-0912 **(B)** and PDO-3890 **(C)** by the qRT–PCR assay following 6-hour treatment with the vehicle control (dark red bars), 0.5 μM of thapsigargin (Tg) alone (orange bars), or 0.5 μM of Tg combined with increasing concentrations of 4μ8C (light yellow bars). The concentration of 4μ8C utilized ranged from 8 to 32 μM in a two-fold gradient. **(D)** The dose-response curves and IC_{50} values of palbociclib in PDO-0912 and PDO-3890 after being treated for 96 h. **(E)** The dose-response curves and IC_{50} values of fulvestrant in PDO-0912 and PDO-3890 after being treated for 96 h. **(F** and **G)** The mRNA levels of E2F1 downstream target genes were assessed in PDO-0912 **(F)** and PDO-3890 **(G)** by the qRT–PCR assay after the indicated treatment for 72 h. PDO-0912 was subjected to the vehicle, a combination of 5 μM palbociclib and 0.5 μM fulvestrant (Pal+Ful), or their combination with 12 μM 4μ8C (Pal+Ful+4μ8C); PDO-3890 was subjected to the vehicle, a combination of 15 μM palbociclib and 1 μM fulvestrant (Pal+Ful), or their combination with 30 μM 4μ8C (Pal+Ful+4μ8C). $*P < 0.05$, $**P < 0.01$, $***P < 0.001$, $****P < 0.0001$. **(H** and **I)** Acridine orange/propidium iodide (AO/PI) staining was performed on two $XBP1s^{high}$ PDOs, PDO-1553 **(H)** and PDO-4576 **(I)** following treatment with the vehicle, 4μ8C, a combination of palbociclib and fulvestrant (Pal+Ful), or their combination with 4μ8C (Pal+Ful+4μ8C). The left panel shows the representative images of live/dead organoids 96 h after the indicated treatment; the right panel shows the quantification results of live (green)/dead (red) analysis. Scale bar, 50 μm. **(J)** Western blotting of indicated proteins from MCF7 cell-derived xenografts in different treatment groups. n = 3

per treatment group. **(K)** Changes in the body weight of mice bearing MCF7 cell-derived xenografts throughout the experiment, starting from day 0 of the treatment to the end (18 days after treatment). n = 6 per treatment group. For statistical analysis, one-way ANOVA with Turkey's post-hoc test was utilized for **F** and **G**. Data are represented as the mean ± standard deviation.

Supplementary Table 1. Predicted binding sites of XBP1s in the SND1 promoter region in the JASPAR database.

Matrix ID	Score	Relative score	Start	End	Strand	Sequence
MA0844.1.XBP1	19.3309	0.9785	1724	1737	-	AAGGACACGTCAGC
MA0844.1.XBP1	4.9719	0.7896	353	366	+	TGGGATAAGTCATG
MA0844.1.XBP1	3.0931	0.7649	1271	1284	-	CATGCCACGTATAC
MA0844.1.XBP1	2.7358	0.7602	1647	1660	+	TGGGCGAGGTCAGG
MA0844.1.XBP1	2.1551	0.7525	436	449	+	AATGACACGGTAGA
MA0844.1.XBP1	1.8515	0.7485	1013	1026	-	TGTGTTATGTCATT
MA0844.1.XBP1	1.3480	0.7419	874	887	+	TGTGTTATGTCATT
MA0844.1.XBP1	1.2255	0.7403	470	483	+	AAAGCCACGAGATA

Supplementary Table 2. Clinicopathological characteristics of patients with metastatic HR+/HER2- breast cancerin the validation cohort (n = 30).

Patient ID	Age at diagnosis with MBC	ER status	PR status	HER2 status	Ki67 (%)	Origin of sample	Adjuvant therapy	Time from surgery to the distant metastases (Months)	Metastatic therapy	Metastatic sites	Number of metastatic sites	PFS	PFS time (Months)	Treatment response group
M-01	60	Positive	Positive	Negative	25	Metastatic	TC, AI	61.7	Fulvestrant+CDK4/6 inhibitor	liver	1	Yes	38.8	Sensitive
M-02	67	Positive	Positive	Negative	15	Metastatic	None (de-nove metastatic)	Not applicable	AI+CDK4/6 inhibitor	bone	1	Yes	15.6	Sensitive
M-03	61	Positive	Positive	Negative	60	Metastatic	None (de-nove metastatic)	Not applicable	Fulvestrant+CDK4/6 inhibitor	bone	1	Yes	14.7	Sensitive
M-04	62	Positive	Negative	Negative	5~10	Metastatic	TAC, AI	42.8	Fulvestrant+CDK4/6 inhibitor	bone	1	Yes	3.3	Resistant
M-05	43	Positive	Positive	Negative	5	Metastatic	CEF-T, TAM	71.8	Fulvestrant+CDK4/6 inhibitor	liver	1	Yes	40.5	Sensitive
M-06	58	Positive	Positive	Negative	50	Metastatic	EC-T, AI	13.8	Fulvestrant+CDK4/6 inhibitor	bone	1	Yes	8.4	Sensitive
M-07	62	Positive	Positive	Negative	40	Metastatic	EC-T, AI	21.6	Fulvestrant+CDK4/6 inhibitor	bone, liver, lymph node	3	Yes	2.5	Resistant
M-08	57	Positive	Positive	Negative	10	Metastatic	None (de-nove metastatic)	Not applicable	AI+CDK4/6 inhibitor	liver, bone	2	No	42.6	Sensitive
M-09	51	Positive	Positive	Negative	30	Metastatic	EC, AI	77.5	AI+CDK4/6 inhibitor	liver, bone	2	Yes	1.8	Resistant
M-10	65	Positive	Negative	Negative	80	Metastatic	TC, AI	33.8	Fulvestrant+CDK4/6 inhibitor	liver, bone	2	Yes	1.6	Resistant
M-11	49	Positive	Positive	Negative	60	Metastatic	TC, TAM	13.3	Fulvestrant+CDK4/6 inhibitor	brain, liver, lymph node	3	Yes	8.5	Sensitive
M-12	49	Positive	Positive	Negative	40	Metastatic	None (de-nove metastatic)	Not applicable	AI+CDK4/6 inhibitor	liver, bone, lymph node	3	Yes	5.1	Resistant
M-13	66	Positive	Negative	Negative	60	Metastatic	TC, AI	21.7	Fulvestrant+CDK4/6 inhibitor	liver, lymph node	2	Yes	2.4	Resistant
M-14	62	Positive	Negative	Negative	15	Metastatic	AI	65.4	Fulvestrant+CDK4/6 inhibitor	liver	1	Yes	9.9	Sensitive
M-15	55	Positive	Positive	Negative	80	Metastatic	TC, AI	52.0	Fulvestrant+CDK4/6 inhibitor	liver	1	Yes	15.5	Sensitive
M-16	59	Positive	Negative	Negative	80	Metastatic	EC-T, AI	14.0	Fulvestrant+CDK4/6 inhibitor	liver	1	No	9.0	Sensitive
M-17	53	Positive	Positive	Negative	30	Metastatic	EC-T, AI	57.8	Fulvestrant+CDK4/6 inhibitor	liver, bone	2	Yes	13.2	Sensitive
M-18	66	Positive	Positive	Negative	30	Metastatic	None (de-nove metastatic)	Not applicable	Fulvestrant+CDK4/6 inhibitor	bone	1	Yes	6.0	Resistant
M-19	61	Positive	Negative	Negative	10	Metastatic	EC-T, AI	37.6	Fulvestrant+CDK4/6 inhibitor	lung, bone	2	Yes	31.8	Sensitive
M-20	46	Positive	Positive	Negative	50	Metastatic	TC, TAM	21.4	Fulvestrant+CDK4/6 inhibitor	liver	1	Yes	21.2	Sensitive
M-21	43	Positive	Negative	Negative	70	Metastatic	AC-T, TAM	22.1	Fulvestrant+CDK4/6 inhibitor	bone	1	Yes	2.4	Resistant
M-22	54	Positive	Positive	Negative	40	Metastatic	EC, AI	32.9	Fulvestrant+CDK4/6 inhibitor	liver	1	Yes	9.0	Sensitive
M-23	32	Positive	Negative	Negative	20	Metastatic	EC-T, AI	39.1	AI+CDK4/6 inhibitor	lung, lymph node	2	Yes	2.1	Resistant
M-24	40	Positive	Positive	Negative	15	Metastatic	None (de-nove metastatic)	Not applicable	AI+CDK4/6 inhibitor	bone, liver, lymph node	3	No	19.5	Sensitive
M-25	50	Positive	Positive	Negative	40	Metastatic	TC	50.2	AI+CDK4/6 inhibitor	lung	1	No	42.6	Sensitive
M-26	54	Positive	Positive	Negative	30	Metastatic	TC, AI	5.9	Fulvestrant+CDK4/6 inhibitor	lung	1	Yes	25.4	Sensitive
M-27	55	Positive	Negative	Negative	10	Metastatic	EC-T, AI	41.6	Fulvestrant+CDK4/6 inhibitor	bone	1	Yes	9.3	Sensitive
M-28	54	Positive	Positive	Negative	10	Metastatic	TC	91.0	Fulvestrant+CDK4/6 inhibitor	liver, bone	2	Yes	21.4	Sensitive
M-29	38	Positive	Positive	Negative	< 5	Metastatic	EC, TAM	86.0	Fulvestrant+CDK4/6 inhibitor	lung, lymph node	2	No	40.5	Sensitive
M-30	63	Positive	Negative	Negative	30	Metastatic	AI	22.7	Fulvestrant+CDK4/6 inhibitor	lung	1	No	29.1	Sensitive

MBC , metastatic breast cancer; *TC* , docetaxel+cyclophosphamide; *TAC* , docetaxel+doxorubin+cyclophosphamide; *CEF-T* , epirubicin+cyclophosphamide+5-fluorouracil-docetaxel; *EC* , epirubicin+cyclophosphamide; *EC-T* , EC-docetaxel; *AI* , aromatase inhibitors; *TAM* , tamoxifen; *PFS* , progression-free survival.

Supplementary Table 3. Clinicopathological characteristics of patients diagnosed with primary breast cancer in FUSCC (n = 274).

Number of patients	Age at diagnosis	Menopause	Histologic subtype	pT stage	pN stage	TNM stage	ER status	PR status	HER2 status	Molecular subtypes
1	52	Yes	Invasive ductal carcinoma	1	0	I	Positive	Positive	Negative	HR-positive/HER2-negative
2	56	Yes	Invasive ductal carcinoma	2	1	II	Positive	Positive	Negative	HR-positive/HER2-negative
3	63	No	Invasive ductal carcinoma	2	0	II	Positive	Negative	Negative	HR-positive/HER2-negative
4	67	Yes	Invasive ductal carcinoma	2	2	III	Positive	Positive	Negative	HR-positive/HER2-negative
5	44	No	Invasive ductal carcinoma	1	0	I	Positive	Positive	Negative	HR-positive/HER2-negative
6	43	No	Invasive ductal carcinoma	2	0	II	Positive	Positive	Positive	HER2-positive
7	57	Yes	Invasive ductal carcinoma	1	1	II	Negative	Negative	Negative	Triple negative breast cancer
8	65	Yes	Invasive ductal carcinoma	2	1	II	Positive	Positive	Negative	HR-positive/HER2-negative
9	34	No	Invasive ductal carcinoma	1	3	III	Negative	Positive	Positive	HER2-positive
10	55	Yes	Invasive ductal carcinoma	2	0	II	Negative	Negative	Negative	Triple negative breast cancer
11	39	No	Invasive ductal carcinoma	2	3	III	Positive	Positive	Negative	HR-positive/HER2-negative
12	48	No	Invasive ductal carcinoma	2	1	II	Positive	Positive	Negative	HR-positive/HER2-negative
13	60	Yes	Invasive ductal carcinoma	1	0	I	Negative	Negative	Negative	Triple negative breast cancer
14	62	Yes	Invasive ductal carcinoma	2	1	II	Positive	Positive	Negative	HR-positive/HER2-negative
15	47	Yes	Invasive ductal carcinoma	2	1	II	Negative	Negative	Negative	Triple negative breast cancer
16	70	Yes	Invasive ductal carcinoma	2	1	II	Positive	Positive	Negative	HR-positive/HER2-negative
17	58	Yes	Invasive ductal carcinoma	1	0	I	Positive	Positive	Negative	HR-positive/HER2-negative
18	33	No	Invasive ductal carcinoma	2	3	III	Positive	Positive	Negative	HR-positive/HER2-negative
19	56	Yes	Invasive ductal carcinoma	2	2	III	Positive	Positive	Negative	HR-positive/HER2-negative
20	53	Yes	Invasive ductal carcinoma	1	0	I	Negative	Positive	Positive	HER2-positive
21	58	Yes	Invasive ductal carcinoma	2	0	II	Negative	Negative	Negative	Triple negative breast cancer
22	58	Yes	Invasive ductal carcinoma	2	3	III	Negative	Negative	Negative	Triple negative breast cancer
23	69	Yes	Invasive ductal carcinoma	2	3	III	Positive	Negative	Negative	HR-positive/HER2-negative
24	52	No	Invasive ductal carcinoma	1	1	II	Positive	Positive	Negative	HR-positive/HER2-negative
25	53	Yes	Invasive ductal carcinoma	2	1	II	Negative	Positive	Negative	HR-positive/HER2-negative
26	54	Yes	Invasive ductal carcinoma	1	0	I	Negative	Negative	Negative	Triple negative breast cancer
27	59	Yes	Invasive ductal carcinoma	1	0	I	Positive	Negative	Negative	HR-positive/HER2-negative
28	44	No	Invasive ductal carcinoma	2	3	III	Negative	Positive	Positive	HER2-positive

Supplementary Table 3. Clinicopathological characteristics of patients diagnosed with primary breast cancer in FUSCC (n = 274).

Number of patients	Age at diagnosis	Menopause	Histologic subtype	pT stage	pN stage	TNM stage	ER status	PR status	HER2 status	Molecular subtypes
29	46	No	Invasive ductal carcinoma	1	0	I	Positive	Positive	Negative	HR-positive/HER2-negative
30	48	No	Invasive ductal carcinoma	2	0	II	Positive	Positive	Positive	HER2-positive
31	87	Yes	Invasive ductal carcinoma	2	3	III	Negative	Positive	Positive	HER2-positive
32	56	Yes	Invasive ductal carcinoma	2	1	II	Positive	Negative	Negative	HR-positive/HER2-negative
33	46	No	Invasive ductal carcinoma	1	0	I	Positive	Positive	Negative	HR-positive/HER2-negative
34	57	Yes	Invasive ductal carcinoma	1	1	II	Positive	Positive	Positive	HER2-positive
35	48	No	Invasive ductal carcinoma	1	2	III	Positive	Positive	Negative	HR-positive/HER2-negative
36	45	No	Invasive ductal carcinoma	2	0	II	Positive	Positive	Negative	HR-positive/HER2-negative
37	53	No	Invasive ductal carcinoma	1	0	I	Positive	Positive	Negative	HR-positive/HER2-negative
38	57	Yes	Invasive ductal carcinoma	2	1	II	Positive	Negative	Positive	HER2-positive
39	58	Yes	Invasive ductal carcinoma	2	0	II	Positive	Positive	Negative	HR-positive/HER2-negative
40	69	Yes	Invasive ductal carcinoma	2	2	III	Negative	Negative	Positive	HER2-positive
41	55	Yes	Invasive ductal carcinoma	2	0	II	Positive	Positive	Positive	HER2-positive
42	82	Yes	Invasive ductal carcinoma	1	0	I	Positive	Positive	Negative	HR-positive/HER2-negative
43	47	No	Invasive ductal carcinoma	2	0	II	Positive	Positive	Negative	HR-positive/HER2-negative
44	56	Yes	Invasive ductal carcinoma	1	1	II	Negative	Negative	Negative	Triple negative breast cancer
45	59	Yes	Invasive ductal carcinoma	2	0	II	Negative	Negative	Negative	Triple negative breast cancer
46	46	No	Invasive ductal carcinoma	2	3	III	Positive	Positive	Negative	HR-positive/HER2-negative
47	59	Yes	Invasive ductal carcinoma	2	0	II	Negative	Negative	Negative	Triple negative breast cancer
48	61	Yes	Invasive ductal carcinoma	2	0	II	Positive	Positive	Negative	HR-positive/HER2-negative
49	30	No	Invasive ductal carcinoma	2	3	III	Positive	Positive	Negative	HR-positive/HER2-negative
50	76	Yes	Invasive ductal carcinoma	1	2	III	Positive	Positive	Negative	HR-positive/HER2-negative
51	56	Yes	Invasive ductal carcinoma	2	2	III	Negative	Negative	Negative	Triple negative breast cancer
52	55	Yes	Invasive ductal carcinoma	2	2	III	Positive	Positive	Negative	HR-positive/HER2-negative
53	59	Yes	Invasive ductal carcinoma	1	1	II	Positive	Positive	Positive	HER2-positive
54	63	Yes	Invasive ductal carcinoma	1	0	I	Positive	Positive	Negative	HR-positive/HER2-negative
55	59	Yes	Invasive ductal carcinoma	1	0	I	Negative	Positive	Positive	HER2-positive
56	58	Yes	Invasive ductal carcinoma	2	0	II	Negative	Negative	Positive	HER2-positive

Supplementary Table 3. Clinicopathological characteristics of patients diagnosed with primary breast cancer in FUSCC (n = 274).

Number of patients	Age at diagnosis	Menopause	Histologic subtype	pT stage	pN stage	TNM stage	ER status	PR status	HER2 status	Molecular subtypes
57	45	No	Invasive ductal carcinoma	2	0	II	Positive	Positive	Positive	HER2-positive
58	43	No	Invasive ductal carcinoma	2	1	II	Positive	Positive	Negative	HR-positive/HER2-negative
59	59	Yes	Invasive ductal carcinoma	2	2	III	Positive	Positive	Negative	HR-positive/HER2-negative
60	40	No	Invasive ductal carcinoma	2	3	III	Positive	Positive	Negative	HR-positive/HER2-negative
61	44	No	Invasive ductal carcinoma	2	1	II	Negative	Positive	Positive	HER2-positive
62	41	No	Invasive ductal carcinoma	1	1	II	Positive	Positive	Negative	HR-positive/HER2-negative
63	46	No	Invasive ductal carcinoma	1	1	II	Positive	Negative	Negative	HR-positive/HER2-negative
64	56	Yes	Invasive ductal carcinoma	2	1	II	Positive	Positive	Positive	HER2-positive
65	62	No	Invasive ductal carcinoma	2	1	II	Positive	Positive	Negative	HR-positive/HER2-negative
66	50	Yes	Invasive ductal carcinoma	2	0	II	Positive	Positive	Negative	HR-positive/HER2-negative
67	41	No	Invasive ductal carcinoma	2	2	III	Positive	Positive	Negative	HR-positive/HER2-negative
68	66	Yes	Invasive ductal carcinoma	1	1	II	Positive	Positive	Negative	HR-positive/HER2-negative
69	31	No	Invasive ductal carcinoma	2	3	III	Positive	Positive	Negative	HR-positive/HER2-negative
70	52	Yes	Invasive ductal carcinoma	1	0	I	Positive	Positive	Negative	HR-positive/HER2-negative
71	61	Yes	Invasive ductal carcinoma	2	0	II	Negative	Negative	Positive	HER2-positive
72	41	No	Invasive ductal carcinoma	2	2	III	Positive	Positive	Positive	HER2-positive
73	60	Yes	Invasive ductal carcinoma	1	1	II	Positive	Positive	Negative	HR-positive/HER2-negative
74	53	Yes	Invasive ductal carcinoma	2	1	II	Positive	Positive	Negative	HR-positive/HER2-negative
75	50	Yes	Invasive ductal carcinoma	1	3	III	Positive	Positive	Negative	HR-positive/HER2-negative
76	85	Yes	Invasive ductal carcinoma	2	3	III	Negative	Negative	Negative	Triple negative breast cancer
77	64	Yes	Invasive ductal carcinoma	1	3	III	Positive	Positive	Negative	HR-positive/HER2-negative
78	57	Yes	Invasive ductal carcinoma	2	3	III	Positive	Positive	Negative	HR-positive/HER2-negative
79	50	No	Invasive ductal carcinoma	1	0	I	Positive	Positive	Positive	HER2-positive
80	55	Yes	Invasive ductal carcinoma	2	3	III	Negative	Positive	Negative	HR-positive/HER2-negative
81	48	No	Invasive ductal carcinoma	2	2	III	Positive	Positive	Negative	HR-positive/HER2-negative
82	63	Yes	Invasive ductal carcinoma	2	3	III	Positive	Positive	Positive	HER2-positive
83	67	Yes	Invasive ductal carcinoma	2	0	II	Positive	Positive	Negative	HR-positive/HER2-negative
84	42	Yes	Invasive ductal carcinoma	2	0	II	Negative	Negative	Positive	HER2-positive

Supplementary Table 3. Clinicopathological characteristics of patients diagnosed with primary breast cancer in FUSCC (n = 274).

Number of patients	Age at diagnosis	Menopause	Histologic subtype	pT stage	pN stage	TNM stage	ER status	PR status	HER2 status	Molecular subtypes
85	64	Yes	Invasive ductal carcinoma	1	0	I	Positive	Positive	Negative	HR-positive/HER2-negative
86	44	No	Invasive ductal carcinoma	2	0	II	Positive	Positive	Positive	HER2-positive
87	47	No	Invasive ductal carcinoma	1	2	III	Negative	Negative	Positive	HER2-positive
88	54	Yes	Invasive ductal carcinoma	2	1	II	Positive	Positive	Negative	HR-positive/HER2-negative
89	63	Yes	Invasive ductal carcinoma	1	1	II	Positive	Positive	Negative	HR-positive/HER2-negative
90	57	Yes	Invasive ductal carcinoma	2	1	II	Negative	Negative	Positive	HER2-positive
91	57	Yes	Invasive ductal carcinoma	2	1	II	Positive	Positive	Negative	HR-positive/HER2-negative
92	57	Yes	Invasive ductal carcinoma	1	1	II	Positive	Positive	Negative	HR-positive/HER2-negative
93	40	No	Invasive ductal carcinoma	2	3	III	Positive	Positive	Negative	HR-positive/HER2-negative
94	58	Yes	Invasive ductal carcinoma	2	1	II	Positive	Positive	Negative	HR-positive/HER2-negative
95	39	No	Invasive ductal carcinoma	1	0	I	Negative	Negative	Negative	Triple negative breast cancer
96	44	No	Invasive ductal carcinoma	2	0	II	Positive	Positive	Negative	HR-positive/HER2-negative
97	54	Yes	Invasive ductal carcinoma	2	0	II	Negative	Negative	Positive	HER2-positive
98	23	No	Invasive ductal carcinoma	2	0	II	Positive	Positive	Negative	HR-positive/HER2-negative
99	61	Yes	Invasive ductal carcinoma	2	0	II	Positive	Positive	Negative	HR-positive/HER2-negative
100	34	No	Invasive ductal carcinoma	1	0	I	Negative	Positive	Negative	HR-positive/HER2-negative
101	54	Yes	Invasive ductal carcinoma	1	0	I	Negative	Negative	Negative	Triple negative breast cancer
102	57	Yes	Invasive ductal carcinoma	2	0	II	Negative	Negative	Negative	Triple negative breast cancer
103	50	No	Invasive ductal carcinoma	2	1	II	Positive	Positive	Negative	HR-positive/HER2-negative
104	44	Yes	Invasive ductal carcinoma	2	1	II	Positive	Positive	Negative	HR-positive/HER2-negative
105	54	Yes	Invasive ductal carcinoma	2	3	III	Negative	Positive	Positive	HER2-positive
106	43	No	Invasive ductal carcinoma	3	1	III	Negative	Negative	Negative	Triple negative breast cancer
107	54	Yes	Invasive ductal carcinoma	1	0	I	Positive	Positive	Negative	HR-positive/HER2-negative
108	44	No	Invasive ductal carcinoma	2	2	III	Positive	Positive	Negative	HR-positive/HER2-negative
109	41	No	Invasive ductal carcinoma	2	0	II	Positive	Positive	Negative	HR-positive/HER2-negative
110	51	No	Invasive ductal carcinoma	1	2	III	Positive	Positive	Negative	HR-positive/HER2-negative
111	63	Yes	Invasive ductal carcinoma	2	3	III	Positive	Positive	Negative	HR-positive/HER2-negative
112	48	No	Invasive ductal carcinoma	2	0	II	Negative	Negative	Positive	HER2-positive

Supplementary Table 3. Clinicopathological characteristics of patients diagnosed with primary breast cancer in FUSCC (n = 274).

Number of patients	Age at diagnosis	Menopause	Histologic subtype	pT stage	pN stage	TNM stage	ER status	PR status	HER2 status	Molecular subtypes
113	63	Yes	Invasive ductal carcinoma	1	0	I	Positive	Positive	Negative	HR-positive/HER2-negative
114	56	Yes	Invasive ductal carcinoma	2	1	II	Negative	Negative	Positive	HER2-positive
115	54	Yes	Invasive ductal carcinoma	2	1	II	Positive	Positive	Negative	HR-positive/HER2-negative
116	47	No	Invasive ductal carcinoma	2	1	II	Negative	Negative	Negative	Triple negative breast cancer
117	47	No	Invasive ductal carcinoma	3	1	III	Positive	Positive	Positive	HER2-positive
118	80	Yes	Invasive ductal carcinoma	2	1	II	Negative	Negative	Negative	Triple negative breast cancer
119	63	Yes	Invasive ductal carcinoma	2	0	II	Positive	Positive	Negative	HR-positive/HER2-negative
120	63	Yes	Invasive ductal carcinoma	2	0	II	Positive	Positive	Negative	HR-positive/HER2-negative
121	63	Yes	Invasive ductal carcinoma	1	0	I	Negative	Negative	Positive	HER2-positive
122	64	Yes	Invasive ductal carcinoma	2	0	II	Positive	Positive	Negative	HR-positive/HER2-negative
123	57	Yes	Invasive ductal carcinoma	2	0	II	Positive	Positive	Negative	HR-positive/HER2-negative
124	36	No	Invasive ductal carcinoma	2	3	III	Negative	Positive	Positive	HER2-positive
125	61	Yes	Invasive ductal carcinoma	2	0	II	Positive	Positive	Positive	HER2-positive
126	54	Yes	Invasive ductal carcinoma	2	3	III	Positive	Positive	Negative	HR-positive/HER2-negative
127	43	No	Invasive ductal carcinoma	2	0	II	Negative	Negative	Positive	HER2-positive
128	61	Yes	Invasive ductal carcinoma	1	0	I	Positive	Positive	Negative	HR-positive/HER2-negative
129	54	No	Invasive ductal carcinoma	2	0	II	Negative	Negative	Negative	Triple negative breast cancer
130	39	No	Invasive ductal carcinoma	2	1	II	Negative	Positive	Positive	HER2-positive
131	50	No	Invasive ductal carcinoma	1	2	III	Negative	Positive	Positive	HER2-positive
132	62	Yes	Invasive ductal carcinoma	2	1	II	Positive	Positive	Negative	HR-positive/HER2-negative
133	52	Yes	Invasive ductal carcinoma	2	2	III	Positive	Positive	Positive	HER2-positive
134	64	Yes	Invasive ductal carcinoma	2	3	III	Positive	Positive	Negative	HR-positive/HER2-negative
135	25	No	Invasive ductal carcinoma	2	1	II	Negative	Positive	Positive	HER2-positive
136	68	Yes	Invasive ductal carcinoma	1	0	I	Negative	Negative	Negative	Triple negative breast cancer
137	52	Yes	Invasive ductal carcinoma	2	1	II	Positive	Positive	Positive	HER2-positive
138	69	Yes	Invasive ductal carcinoma	1	0	I	Positive	Positive	Negative	HR-positive/HER2-negative
139	38	No	Invasive ductal carcinoma	2	0	II	Negative	Negative	Negative	Triple negative breast cancer
140	44	No	Invasive ductal carcinoma	1	0	I	Positive	Positive	Negative	HR-positive/HER2-negative

Supplementary Table 3. Clinicopathological characteristics of patients diagnosed with primary breast cancer in FUSCC (n = 274).

Number of patients	Age at diagnosis	Menopause	Histologic subtype	pT stage	pN stage	TNM stage	ER status	PR status	HER2 status	Molecular subtypes
141	60	Yes	Invasive ductal carcinoma	2	2	III	Negative	Negative	Positive	HER2-positive
142	70	Yes	Invasive ductal carcinoma	1	0	I	Positive	Positive	Negative	HR-positive/HER2-negative
143	45	No	Invasive ductal carcinoma	2	2	III	Positive	Positive	Negative	HR-positive/HER2-negative
144	61	Yes	Invasive ductal carcinoma	1	3	III	Positive	Positive	Negative	HR-positive/HER2-negative
145	66	Yes	Invasive ductal carcinoma	2	1	II	Negative	Positive	Positive	HER2-positive
146	49	No	Invasive ductal carcinoma	2	3	III	Positive	Positive	Negative	HR-positive/HER2-negative
147	36	No	Invasive ductal carcinoma	3	3	III	Positive	Positive	Negative	HR-positive/HER2-negative
148	54	Yes	Invasive ductal carcinoma	3	3	III	Negative	Negative	Negative	Triple negative breast cancer
149	46	No	Invasive ductal carcinoma	2	1	II	Negative	Negative	Negative	Triple negative breast cancer
150	79	Yes	Invasive ductal carcinoma	2	0	II	Negative	Positive	Negative	HR-positive/HER2-negative
151	47	Yes	Invasive ductal carcinoma	2	0	II	Positive	Positive	Negative	HR-positive/HER2-negative
152	58	Yes	Invasive ductal carcinoma	1	0	I	Negative	Negative	Positive	HER2-positive
153	47	No	Invasive ductal carcinoma	2	0	II	Positive	Positive	Positive	HER2-positive
154	62	Yes	Invasive ductal carcinoma	1	1	II	Positive	Positive	Positive	HER2-positive
155	54	Yes	Invasive ductal carcinoma	2	3	III	Negative	Negative	Negative	Triple negative breast cancer
156	62	Yes	Invasive ductal carcinoma	1	0	I	Negative	Negative	Negative	Triple negative breast cancer
157	60	Yes	Invasive ductal carcinoma	3	1	III	Positive	Positive	Negative	HR-positive/HER2-negative
158	30	No	Invasive ductal carcinoma	2	1	II	Positive	Positive	Negative	HR-positive/HER2-negative
159	42	No	Invasive ductal carcinoma	2	1	II	Positive	Positive	Negative	HR-positive/HER2-negative
160	57	Yes	Invasive ductal carcinoma	2	1	II	Positive	Positive	Positive	HER2-positive
161	45	No	Invasive ductal carcinoma	2	0	II	Positive	Positive	Positive	HER2-positive
162	56	Yes	Invasive ductal carcinoma	1	1	II	Positive	Positive	Negative	HR-positive/HER2-negative
163	61	Yes	Invasive ductal carcinoma	1	0	I	Negative	Positive	Positive	HER2-positive
164	76	Yes	Invasive ductal carcinoma	2	1	II	Positive	Positive	Negative	HR-positive/HER2-negative
165	49	Yes	Invasive ductal carcinoma	1	0	I	Positive	Positive	Positive	HER2-positive
166	42	No	Invasive ductal carcinoma	1	1	II	Positive	Positive	Negative	HR-positive/HER2-negative
167	45	No	Invasive ductal carcinoma	2	0	II	Negative	Negative	Positive	HER2-positive
168	61	Yes	Invasive ductal carcinoma	1	0	I	Positive	Positive	Negative	HR-positive/HER2-negative

Supplementary Table 3. Clinicopathological characteristics of patients diagnosed with primary breast cancer in FUSCC (n = 274).

Number of patients	Age at diagnosis	Menopause	Histologic subtype	pT stage	pN stage	TNM stage	ER status	PR status	HER2 status	Molecular subtypes
169	42	Yes	Invasive ductal carcinoma	2	2	III	Positive	Positive	Negative	HR-positive/HER2-negative
170	85	Yes	Invasive ductal carcinoma	2	3	III	Negative	Negative	Negative	Triple negative breast cancer
171	37	No	Invasive ductal carcinoma	3	1	III	Negative	Positive	Positive	HER2-positive
172	49	No	Invasive ductal carcinoma	2	0	II	Negative	Negative	Positive	HER2-positive
173	51	Yes	Invasive ductal carcinoma	2	2	III	Positive	Positive	Positive	HER2-positive
174	47	No	Invasive ductal carcinoma	1	0	I	Negative	Negative	Positive	HER2-positive
175	51	No	Invasive ductal carcinoma	1	0	I	Negative	Positive	Positive	HER2-positive
176	42	No	Invasive ductal carcinoma	2	0	II	Positive	Positive	Negative	HR-positive/HER2-negative
177	51	No	Invasive ductal carcinoma	2	0	II	Positive	Positive	Negative	HR-positive/HER2-negative
178	61	Yes	Invasive ductal carcinoma	2	1	II	Negative	Negative	Negative	Triple negative breast cancer
179	52	Yes	Invasive ductal carcinoma	2	0	II	Negative	Negative	Negative	Triple negative breast cancer
180	60	Yes	Invasive ductal carcinoma	2	1	II	Negative	Positive	Positive	HER2-positive
181	73	Yes	Invasive ductal carcinoma	1	1	II	Positive	Positive	Negative	HR-positive/HER2-negative
182	57	Yes	Invasive ductal carcinoma	2	1	II	Positive	Positive	Negative	HR-positive/HER2-negative
183	58	Yes	Invasive ductal carcinoma	1	0	I	Positive	Positive	Negative	HR-positive/HER2-negative
184	80	Yes	Invasive ductal carcinoma	1	1	II	Negative	Positive	Negative	HR-positive/HER2-negative
185	54	No	Invasive ductal carcinoma	1	0	I	Positive	Positive	Negative	HR-positive/HER2-negative
186	78	Yes	Invasive ductal carcinoma	1	1	II	Negative	Positive	Negative	HR-positive/HER2-negative
187	80	Yes	Invasive ductal carcinoma	1	3	III	Positive	Positive	Negative	HR-positive/HER2-negative
188	62	No	Invasive ductal carcinoma	2	0	II	Positive	Positive	Negative	HR-positive/HER2-negative
189	60	Yes	Invasive ductal carcinoma	3	0	II	Positive	Positive	Positive	HER2-positive
190	76	Yes	Invasive ductal carcinoma	2	3	III	Positive	Positive	Positive	HER2-positive
191	49	No	Invasive ductal carcinoma	2	0	II	Negative	Negative	Negative	Triple negative breast cancer
192	47	No	Invasive ductal carcinoma	1	0	I	Negative	Negative	Negative	Triple negative breast cancer
193	55	Yes	Invasive ductal carcinoma	2	0	II	Negative	Positive	Negative	HR-positive/HER2-negative
194	47	No	Invasive ductal carcinoma	2	3	III	Negative	Negative	Negative	Triple negative breast cancer
195	71	Yes	Invasive ductal carcinoma	2	0	II	Positive	Negative	Positive	HER2-positive
196	52	No	Invasive ductal carcinoma	1	2	III	Positive	Positive	Negative	HR-positive/HER2-negative

Supplementary Table 3. Clinicopathological characteristics of patients diagnosed with primary breast cancer in FUSCC (n = 274).

Number of patients	Age at diagnosis	Menopause	Histologic subtype	pT stage	pN stage	TNM stage	ER status	PR status	HER2 status	Molecular subtypes
197	53	Yes	Invasive ductal carcinoma	1	0	I	Positive	Positive	Negative	HR-positive/HER2-negative
198	52	No	Invasive ductal carcinoma	2	1	II	Negative	Positive	Positive	HER2-positive
199	52	Yes	Invasive ductal carcinoma	1	1	II	Positive	Positive	Negative	HR-positive/HER2-negative
200	60	Yes	Invasive ductal carcinoma	1	0	I	Positive	Positive	Negative	HR-positive/HER2-negative
201	43	No	Invasive ductal carcinoma	2	0	II	Negative	Negative	Negative	Triple negative breast cancer
202	50	No	Invasive ductal carcinoma	1	0	I	Negative	Negative	Positive	HER2-positive
203	53	Yes	Invasive ductal carcinoma	1	0	I	Positive	Positive	Positive	HER2-positive
204	51	Yes	Invasive ductal carcinoma	2	1	II	Positive	Negative	Positive	HER2-positive
205	40	No	Invasive ductal carcinoma	3	0	II	Positive	Positive	Negative	HR-positive/HER2-negative
206	42	No	Invasive ductal carcinoma	2	0	II	Positive	Positive	Negative	HR-positive/HER2-negative
207	28	No	Invasive ductal carcinoma	1	2	III	Positive	Positive	Negative	HR-positive/HER2-negative
208	52	Yes	Invasive ductal carcinoma	1	1	II	Positive	Positive	Negative	HR-positive/HER2-negative
209	59	Yes	Invasive ductal carcinoma	1	3	III	Positive	Positive	Negative	HR-positive/HER2-negative
210	36	No	Invasive ductal carcinoma	2	0	II	Positive	Positive	Negative	HR-positive/HER2-negative
211	33	No	Invasive ductal carcinoma	1	0	I	Positive	Positive	Positive	HER2-positive
212	59	Yes	Invasive ductal carcinoma	2	1	II	Positive	Positive	Negative	HR-positive/HER2-negative
213	55	Yes	Invasive ductal carcinoma	2	1	II	Positive	Positive	Negative	HR-positive/HER2-negative
214	31	No	Invasive ductal carcinoma	2	2	III	Negative	Negative	Positive	HER2-positive
215	63	Yes	Invasive ductal carcinoma	1	1	II	Positive	Positive	Negative	HR-positive/HER2-negative
216	47	No	Invasive ductal carcinoma	2	0	II	Positive	Positive	Negative	HR-positive/HER2-negative
217	50	Yes	Invasive ductal carcinoma	1	3	III	Positive	Positive	Negative	HR-positive/HER2-negative
218	43	Yes	Invasive ductal carcinoma	2	3	III	Positive	Positive	Negative	HR-positive/HER2-negative
219	69	Yes	Invasive ductal carcinoma	1	1	II	Positive	Positive	Negative	HR-positive/HER2-negative
220	57	Yes	Invasive ductal carcinoma	2	1	II	Positive	Positive	Negative	HR-positive/HER2-negative
221	35	No	Invasive ductal carcinoma	2	3	III	Positive	Positive	Negative	HR-positive/HER2-negative
222	56	Yes	Invasive ductal carcinoma	1	1	II	Positive	Positive	Negative	HR-positive/HER2-negative
223	40	No	Invasive ductal carcinoma	1	3	III	Positive	Positive	Negative	HR-positive/HER2-negative
224	49	No	Invasive ductal carcinoma	1	1	II	Negative	Positive	Positive	HER2-positive

Supplementary Table 3. Clinicopathological characteristics of patients diagnosed with primary breast cancer in FUSCC (n = 274).

Number of patients	Age at diagnosis	Menopause	Histologic subtype	pT stage	pN stage	TNM stage	ER status	PR status	HER2 status	Molecular subtypes
225	48	No	Invasive ductal carcinoma	1	0	I	Positive	Positive	Negative	HR-positive/HER2-negative
226	55	Yes	Invasive ductal carcinoma	2	2	III	Negative	Positive	Positive	HER2-positive
227	71	Yes	Invasive ductal carcinoma	1	3	III	Positive	Positive	Negative	HR-positive/HER2-negative
228	29	No	Invasive ductal carcinoma	2	1	II	Positive	Positive	Negative	HR-positive/HER2-negative
229	43	No	Invasive ductal carcinoma	2	2	III	Positive	Positive	Positive	HER2-positive
230	58	Yes	Invasive ductal carcinoma	1	0	I	Positive	Positive	Negative	HR-positive/HER2-negative
231	46	No	Invasive ductal carcinoma	2	1	II	Positive	Positive	Positive	HER2-positive
232	81	Yes	Invasive ductal carcinoma	1	0	I	Positive	Positive	Positive	HER2-positive
233	42	No	Invasive ductal carcinoma	1	0	I	Negative	Positive	Positive	HER2-positive
234	45	No	Invasive ductal carcinoma	2	1	II	Positive	Positive	Negative	HR-positive/HER2-negative
235	44	No	Invasive ductal carcinoma	2	0	II	Positive	Positive	Negative	HR-positive/HER2-negative
236	53	Yes	Invasive ductal carcinoma	2	1	II	Negative	Negative	Negative	Triple negative breast cancer
237	40	No	Invasive ductal carcinoma	2	2	III	Positive	Positive	Negative	HR-positive/HER2-negative
238	57	Yes	Invasive ductal carcinoma	1	0	I	Positive	Positive	Positive	HER2-positive
239	60	Yes	Invasive ductal carcinoma	2	1	II	Positive	Positive	Negative	HR-positive/HER2-negative
240	48	No	Invasive ductal carcinoma	1	0	I	Positive	Positive	Negative	HR-positive/HER2-negative
241	55	Yes	Invasive ductal carcinoma	2	1	II	Negative	Negative	Negative	Triple negative breast cancer
242	79	Yes	Invasive ductal carcinoma	2	1	II	Positive	Positive	Negative	HR-positive/HER2-negative
243	45	No	Invasive ductal carcinoma	2	0	II	Positive	Positive	Positive	HER2-positive
244	42	No	Invasive ductal carcinoma	2	2	III	Positive	Positive	Negative	HR-positive/HER2-negative
245	55	Yes	Invasive ductal carcinoma	2	1	II	Positive	Positive	Negative	HR-positive/HER2-negative
246	43	No	Invasive ductal carcinoma	2	1	II	Positive	Positive	Negative	HR-positive/HER2-negative
247	69	Yes	Invasive ductal carcinoma	2	0	II	Positive	Positive	Positive	HER2-positive
248	57	Yes	Invasive ductal carcinoma	1	0	I	Positive	Positive	Positive	HER2-positive
249	45	No	Invasive ductal carcinoma	2	2	III	Positive	Positive	Negative	HR-positive/HER2-negative
250	53	Yes	Invasive ductal carcinoma	1	0	I	Negative	Negative	Positive	HER2-positive
251	57	Yes	Invasive ductal carcinoma	3	0	II	Positive	Positive	Positive	HER2-positive
252	56	Yes	Invasive ductal carcinoma	2	2	III	Positive	Negative	Positive	HER2-positive

Supplementary Table 3. Clinicopathological characteristics of patients diagnosed with primary breast cancer in FUSCC (n = 274).

Number of patients	Age at diagnosis	Menopause	Histologic subtype	pT stage	pN stage	TNM stage	ER status	PR status	HER2 status	Molecular subtypes
253	54	No	Invasive ductal carcinoma	2	1	II	Negative	Positive	Negative	HR-positive/HER2-negative
254	68	Yes	Invasive ductal carcinoma	1	0	I	Positive	Positive	Negative	HR-positive/HER2-negative
255	54	Yes	Invasive ductal carcinoma	3	0	II	Positive	Positive	Negative	HR-positive/HER2-negative
256	41	No	Invasive ductal carcinoma	3	2	III	Positive	Positive	Positive	HER2-positive
257	69	Yes	Invasive ductal carcinoma	2	3	III	Positive	Positive	Negative	HR-positive/HER2-negative
258	60	Yes	Invasive ductal carcinoma	1	0	I	Negative	Negative	Negative	Triple negative breast cancer
259	77	Yes	Invasive ductal carcinoma	2	0	II	Positive	Positive	Negative	HR-positive/HER2-negative
260	69	Yes	Invasive ductal carcinoma	1	3	III	Positive	Positive	Negative	HR-positive/HER2-negative
261	49	No	Invasive ductal carcinoma	1	1	II	Positive	Positive	Positive	HER2-positive
262	61	No	Invasive ductal carcinoma	2	0	II	Positive	Negative	Positive	HER2-positive
263	56	Yes	Invasive ductal carcinoma	2	3	III	Negative	Negative	Positive	HER2-positive
264	57	Yes	Invasive ductal carcinoma	1	1	II	Positive	Positive	Negative	HR-positive/HER2-negative
265	61	Yes	Invasive ductal carcinoma	1	0	I	Negative	Negative	Negative	Triple negative breast cancer
266	57	Yes	Invasive ductal carcinoma	1	0	I	Negative	Negative	Positive	HER2-positive
267	62	Yes	Invasive ductal carcinoma	2	3	III	Positive	Positive	Positive	HER2-positive
268	50	No	Invasive ductal carcinoma	2	0	II	Negative	Negative	Negative	Triple negative breast cancer
269	48	Yes	Invasive ductal carcinoma	2	1	II	Positive	Negative	Positive	HER2-positive
270	62	Yes	Invasive ductal carcinoma	2	0	II	Positive	Positive	Negative	HR-positive/HER2-negative
271	51	No	Invasive ductal carcinoma	2	0	II	Positive	Positive	Negative	HR-positive/HER2-negative
272	68	Yes	Invasive ductal carcinoma	1	1	II	Positive	Positive	Negative	HR-positive/HER2-negative
273	40	No	Invasive ductal carcinoma	1	0	I	Positive	Positive	Positive	HER2-positive
274	45	No	Invasive ductal carcinoma	2	3	III	Negative	Negative	Negative	Triple negative breast cancer

FUSCC , Fudan University Shanghai Cancer Center; *pT stage* , pathological tumor stage; *pN stage* , pathological lymph node; *ER* , estrogen receptor; *PR* , progesterone receptor; *HER2* , human epidermal growth factor receptor 2.

Supplementary Table 4. The sequences of primers used in this study.

Name	Sequence	
	Forward	Reverse
**Primers for qRT-PCR**		
TMEM26	GCAGTTTCCACTTGACCTGGCA	GAAGACGCTGATTCCGATGTTCC
ACTIN (ACTB)	CACCATTGGCAATGAGCGGTTC	AGGTCTTTGCGGATGTCCACGT
spliced XBP1 (XBP1s)	GGTCTGCTGAGTCCGCAGCAGG	GGGCTTGGTATATATGTGG
unspliced XBP1 (XBP1u)	CTGCCAGAGATCGAAAGAAGGC	CTCCTGGTTCTCAACTACAAGGC
DNAJB9	GCCATGAAGTACCACCCTGACA	TCGTCTATTAGCATCTGAGAGTGT
HSPA5	CTGTCCAGGCTGGTGTGCTCT	CTTGGTAGGCACCACTGTGTTC
DNAJC3	GGAGAGGATTTGCCACTGCTTTT	CTCTGCTCGATCTTTCAGGGCA
HERPUD1	CCAATGTCTCAGGGACTTGCTTC	CGATTAGAACCAGCAGGCTCCT
DERL1	CCCAATGGACTTGGGAGGAAGA	GGCACACCAAATCCTGATACTCC
EDEM1	ACGAGCAGTGAAAGCCCTTTGG	CCACTCTGCTTTCCAACCCAGT
SND1	GGTGGACTACATTAGACCAGCC	AGACCTTTGCTGACAAGAGCCTC
RRM2	CTGGCTCAAGAAACGAGGACTG	CTCTCCTCCGATGGTTTGTGTAC
POLD3	TGAGCCTGAACCTCCTTCTGTC	CTGTGCAGGATTCACTCTCGTAG
PCNA	CAAGTAATGTCGATAAAGAGGAGG	GTGTCACCGTTGAAGAGAGTGG
MYC	CCTGGTGCTCCATGAGGAGAC	CAGACTCTGACCTTTTGCCAGG
METTL4	CTTGGTCTGTGGAGGTAGTTGC	CCAGTATAAGACCTTCGTAGGGC
E2F1	GGACCTGGAAACTGACCATCAG	CAGTGAGGTCTCATAGCGTGAC
EZH2	GACCTCTGTCTTACTTGTGGAGC	CGTCAGATGGTGCCAGCAATAG
CCNE1	TGTGTCCTGGATGTTGACTGCC	CTCTATGTCGCACCACTGATACC
CKS2	GAGGAGACTTGGTGTCCAACAG	GATTTGACGATCCCCAGATAAACT
CCNA2	CTCTACACAGTCACGGGACAAAG	CTGTGGTGCTTTGAGGTAGGTC
**Primers for ChIP-qPCR**		
SND1	CCAGATGCTGACGTGTCCTT	ATGGGCTAAATATGGGGCGG
**Primers for plasmid construction**		
XBP1	ATTCTAGAGCTAGCGAATTCATGGTGG TGGTGGCAGCCGCG	ATGGTCTTTGTAGTCGGATCCTTAGTT CATTAATGGCTTCCAGC
XBP1s	ATTCTAGAGCTAGCGAATTCATGGTGG TGGTGGCAGCCGCG	(without terminate code) ATGGTCTTTGTAGTCGGATCCGACACT AATCAGCTGGGGAAAGAGTTCAT
		(with terminate code) ATGGTCTTTGTAGTCGGATCCTTAGAC ACTAATCAGCTGGGGAAAGAGTTCAT
GFP	CGCGGATCCATGGTGAGCAAGGGCG	CGCGGATCCTTACTTGTACAGCTCGTC
SND1	ATTCTAGAGCTAGCGAATTCATGGCGT CCTCCGCGCA	ATGGTCTTTGTAGTCGGATCCGCGGCT GTAGCCAAATTCGT

Supplementary Table 4. The sequences of primers used in this study.

Name	Sequence	
	Forward	Reverse
sh-NC	ccggCAACAAGATGAAGAGCACCAActcgagTTGGTGCTCTTCATCTTGTTGtttttg	aattcaaaaaCAACAAGATGAAGAGCACCAActcgagTTGGTGCTCTTCATCTTGTTG
XBP1-sh1	ccggGGTTGAGAACCAGGAGTTAAGctcgagCTTAACTCCTGGTTCTCAACCtttttg	aattcaaaaaGGTTGAGAACCAGGAGTTAAGctcgagCTTAACTCCTGGTTCTCAACC
XBP1-sh2	ccggGACCCAGTCATGTTCTTCAAActcgagTTTGAAGAACATGACTGGGTCtttttg	aattcaaaaaGACCCAGTCATGTTCTTCAAActcgagTTTGAAGAACATGACTGGGTC
SND1-sh1	ccggCCCACAGCTAATTTGGACCAActcgagTTGGTCCAAATTAGCTGTGGGtttttg	aattcaaaaaCCCACAGCTAATTTGGACCAActcgagTTGGTCCAAATTAGCTGTGGG
SND1-sh2	ccggGCGAGAGTATGGCATGATCTActcgagTAGATCATGCCATACTCTCGCtttttg	aattcaaaaaGCGAGAGTATGGCATGATCTActcgagTAGATCATGCCATACTCTCGC
SND1-sh3	ccggGCCAAAGGAAACTTGCCTTATctcgagATAAGGCAAGTTTCCTTTGGCtttttg	aattcaaaaaGCCAAAGGAAACTTGCCTTATctcgagATAAGGCAAGTTTCCTTTGGC
SND1-promoter	CCAGAACATTTCTCTATCGATAGGTACTACAAGTGTGCGCCCCTGA	ACTTAGATCGCAGATCTCGACCAGGAAGCGCTGAAAGAG
SND1-promoter-MUT	GCCAGATGCTATTTGGTCCTTTCCTTC	GAAGGAAAGGACCAAATAGCATCTGGC

Supplementary Table 5. The components for the breast cancer organoid culture medium.

Media component	Source	Catalogue number	Final concentration
R-Spondin 1	Peprotech	120-38	500 $ng·ml^{-1}$
FGF 7	Peprotech	100-19	5 $ng·ml^{-1}$
FGF 10	Peprotech	100-26	20 $ng·ml^{-1}$
EGF	Gibco	PHG0311	5 $ng·ml^{-1}$
Neuregulin 1	Peprotech	100-03	5 nM
Noggin	Peprotech	120-10C	100 $ng·ml^{-1}$
A83-01	Sigma	SML0788	500 nM
Y-27632	Selleck	S1049	5 mM
SB202190	Sigma	S7067	500 nM
B27 supplement	Gibco	17504-44	1x
N-Acetylcysteine	Sigma	A9165-5g	1.25 mM
Nicotinamide	Sigma	N0636	5 mM
β-estradiol	Sigma	E8875	0.5 nM
GlutaMax 100x	Invitrogen	12634-034	1x
Hepes	Invitrogen	15630-056	10 mM
Primocin	Invivogen	Ant-pm-2	1x
Advanced DMEM/F12	Invitrogen	12634-034	1x

Supplementary Table 6. Clinicopathological characteristics of patient-derived organoids (PDOs) used in this study.

PDO ID	Histologic subtype	ER status	PR status	HER2 status	Ki67 (%)
PDO-2042	Invasive ductal carcinoma	Positive	Positive	Negative	< 10
PDO-3874	Invasive ductal carcinoma	Positive	Positive	Negative	10
PDO-7601	Invasive ductal carcinoma	Positive	Positive	Negative	20
PDO-8771	Invasive ductal carcinoma	Positive	Positive	Negative	30
PDO-8862	Invasive ductal carcinoma	Positive	Positive	Negative	60
PDO-9350	Invasive ductal carcinoma	Positive	Positive	Negative	10
PDO-1087	Invasive ductal carcinoma	Positive	Positive	Negative	10
PDO-1553	Invasive ductal carcinoma	Positive	Positive	Negative	15
PDO-4576	Invasive ductal carcinoma	Positive	Positive	Negative	30
PDO-6259	Invasive ductal carcinoma	Positive	Positive	Negative	20
PDO-7611	Invasive ductal carcinoma	Positive	Positive	Negative	40
PDO-8901	Invasive ductal carcinoma	Positive	Positive	Negative	10
PDO-8912	Invasive ductal carcinoma	Positive	Negative	Negative	60
PDO-3890	Invasive ductal carcinoma	Positive	Negative	Negative	60
PDO-0912	Invasive ductal carcinoma	Positive	Positive	Negative	30

ER , estrogen receptor; *PR* , progesterone receptor; *HER2* , human epidermal growth factor receptor 2.
